# Transcription Inhibitors with XRE DNA-Binding and Cupin Signal-Sensing Domains Drive Metabolic Diversification in *Pseudomonas*

**DOI:** 10.1128/mSystems.00753-20

**Published:** 2021-01-12

**Authors:** Julian Trouillon, Michel Ragno, Victor Simon, Ina Attrée, Sylvie Elsen

**Affiliations:** a Université Grenoble Alpes, Bacterial Pathogenesis and Cellular Responses team, CNRS ERL5261, CEA IRIG-BCI, INSERM UMR1036, Grenoble, France; Leiden University

**Keywords:** *Pseudomonas aeruginosa*, transcription factors, XRE-cupin, RNA-seq, DAP-seq, global regulatory networks

## Abstract

Transcription factors (TFs) are instrumental in the bacterial response to new environmental conditions. They can act as direct signal sensors and subsequently induce changes in gene expression leading to physiological adaptation. Here, by combining transcriptome sequencing (RNA-seq) and cistrome determination (DAP-seq), we studied a family of eight TFs in Pseudomonas aeruginosa. This family, encompassing TFs with XRE-like DNA-binding and cupin signal-sensing domains, includes the metabolic regulators ErfA, PsdR, and PauR and five so-far-unstudied TFs. The genome-wide delineation of their regulons identified 39 regulatory interactions with genes mostly involved in metabolism. We found that the XRE-cupin TFs are inhibitors of their neighboring genes, forming local, functional units encoding proteins with functions in condition-specific metabolic pathways. Growth phenotypes of isogenic mutants highlighted new roles for PauR and PA0535 in polyamines and arginine metabolism. The phylogenetic analysis of this family of regulators across the bacterial kingdom revealed a wide diversity of such metabolic regulatory modules and identified species with potentially higher metabolic versatility. Numerous genes encoding uncharacterized XRE-cupin TFs were found near metabolism-related genes, illustrating the need of further systematic characterization of transcriptional regulatory networks in order to better understand the mechanisms of bacterial adaptation to new environments.

**IMPORTANCE** Bacteria of the *Pseudomonas* genus, including the major human pathogen Pseudomonas aeruginosa, are known for their complex regulatory networks and high number of transcription factors, which contribute to their impressive adaptive ability. However, even in the most studied species, most of the regulators are still uncharacterized. With the recent advances in high-throughput sequencing methods, it is now possible to fill this knowledge gap and help the understanding of how bacteria adapt and thrive in new environments. By leveraging these methods, we provide an example of a comprehensive analysis of an entire family of transcription factors and bring new insights into metabolic and regulatory adaptation in the *Pseudomonas* genus.

## Abstract (precis)

Bacteria of the *Pseudomonas* genus, including the major human pathogen Pseudomonas aeruginosa, are known for their complex regulatory networks and high number of transcription factors, which contribute to their impressive adaptive ability. However, even in the most studied species, most of the regulators are still uncharacterized.

## INTRODUCTION

The regulation of gene transcription is a key mechanism in the evolution and adaptation of bacteria to a wide array of environments. Transcriptional regulation involves the interaction between *trans*-acting regulatory proteins called transcription factors (TFs) and *cis*-regulatory elements found on the promoters of regulated genes. Many external and internal signals, such as metabolite concentrations, oxygen, temperature, pH levels, or surface contact, are integrated into regulatory networks through different sensing mechanisms in order to provide an appropriate transcriptional response allowing the bacterium to adapt effectively to the new environment. TFs are classified in several families depending on the structural similarities in their DNA-binding domains (DBDs), which can either stand alone or be present in multidomain proteins containing variable arrangements with sensing or oligomerization domains (1). This allows the integration of countless different input and output signals into regulatory networks. Although TFs are classified in defined families (2), they each control specific sets of genes to coordinate bacterial responses, and the targets and functions of each newly identified TF cannot be inferred but need to be experimentally established. One-component systems (OCSs) are the most diverse TFs that can directly sense intracellular or imported extracellular signals through their signal-sensing domain and transmit the signal, probably through a conformational change, to their DBD (1). Here, we explored one family of OCSs in Pseudomonas aeruginosa, a Gram-negative opportunistic human pathogen.

P. aeruginosa possesses a considerable metabolic versatility and one of the most complex regulatory networks found in the bacterial kingdom, with roughly 10% of all genes dedicated to transcription regulation (3–5). This regulatory network contains about 500 predicted TFs. While some regulatory pathways have been thoroughly studied, the vast majority of P. aeruginosa TFs are still uncharacterized. With the development of next-generation sequencing (NGS)-based methods dedicated to the genome-wide characterization of TFs, the view of complex interplays between and inside different families of TFs is starting to emerge (6, 7). We recently characterized a transcriptional inhibitor, ErfA, recruited to downregulate the expression of a horizontally transferred operon encoding a major virulence factor, ExlA, specifically in one P. aeruginosa lineage (8). ErfA belongs to the large family of regulators with an XRE-like DBD that usually binds their DNA targets as homodimers. Besides its N-terminal DBD, ErfA possesses a C-terminal putative sensor domain with a “cupin” fold (8). While ErfA was rewired to also regulate the horizontally acquired *exlBA* operon in P. aeruginosa, its main conserved target across *Pseudomonas* species is a metabolic operon, located adjacent to the *erfA* gene, with no role in bacterial virulence. ErfA could thus act as a sensory switch in response to yet-unknown conditions. While signal-sensing TFs are widely distributed in bacteria (1), TFs sharing ErfA-specific domain architecture have not been studied, raising the question of whether ErfA-like regulators could be common and important in metabolic and/or virulence regulation.

In this work, we focused on the eight P. aeruginosa TFs that share ErfA architecture in order to examine their genome-wide regulatory targets. In addition to ErfA (8), the family comprises two members with incompletely defined regulons and five uncharacterized TFs. The combination of transcriptome sequencing (RNA-seq) and cistrome determination (DAP-seq) approaches allowed us to define the regulons of six of these TFs. Each family member has specific targets ranging from one to 12 binding sites in promoters of genes related to small-molecule uptake or processing. Regulators with XRE-cupin domains were found as local, specialized inhibitors that are widespread across the *Pseudomonas* genus. While many uncharacterized XRE-cupin TFs were identified, some species (such as Pseudomonas putida) harbored up to 10 of these regulators whereas others (i.e., Pseudomonas stutzeri) possess only one, potentially reflecting different metabolic versatilities.

## RESULTS

### Eight TFs of the XRE-like family share similar architectures.

**FIG 1:**
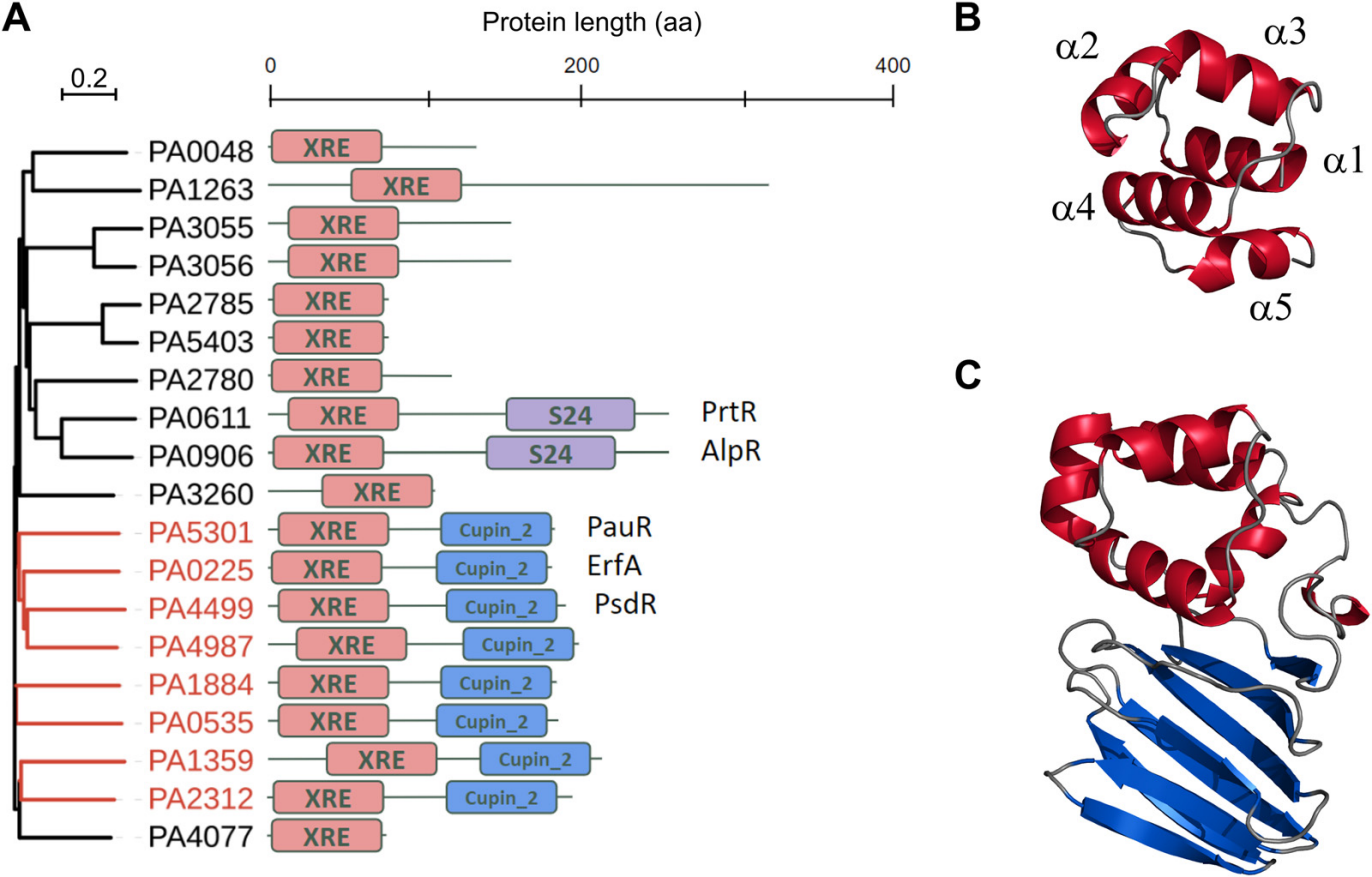
P. aeruginosa TFs belonging to the XRE family. (A) Maximum likelihood phylogenetic tree of the 19 proteins possessing an XRE DNA-binding domain in P. aeruginosa PAO1 (18). The branches corresponding to the eight XRE-cupin regulators are colored in red in the tree. (B) Stereo ribbon representation of the model of the XRE DNA-binding domain of ErfA. The five alpha-helices are annotated. (C) Stereo ribbon representation of the model of ErfA structure. The XRE domain is in red and the cupin domain is in blue. Model prediction was done using the SWISS-MODEL tool (34) using the structure with PDB ID 1Y9Q as the template.

The P. aeruginosa PAO1 genome encodes 19 proteins with a helix-turn-helix DNA-binding motif similar to that of the CI and Cro repressors of the phage λ that features the members of the XRE-like family (9, 10) (Fig. 1A). The XRE-like domains of about 60 residues adopt a well-characterized helical conformation with helices 2 and 3 involved in DNA binding (Fig. 1B). The DBD was found alone or associated either with a peptidase S24 domain (AlpR and PrtR) or with a predicted sensor cupin domain at the C terminus of proteins (Fig. 1C). The versatile cupin domain folds into a 6-stranded β-barrel and is associated with a wide range of function (11). In addition to ErfA (PA0225), seven TFs share the XRE-cupin architecture with only two having partially attributed functions: PA0535, PA1359, PA1884, PA2312, PA4499 (PsdR), PA4987, and PA5301 (PauR). Using targeted approaches, PsdR and PauR were previously shown to regulate dipeptide metabolism (12, 13) and polyamine metabolism (14), respectively. By regulating independent metabolic pathways, these two TFs play an essential role for bacterial growth in their corresponding environmental conditions, which predicts potentially important functions for the five so-far-unstudied TFs of the family.

### TFs of the XRE-cupin family are local, highly specialized repressors.

**FIG 2:**
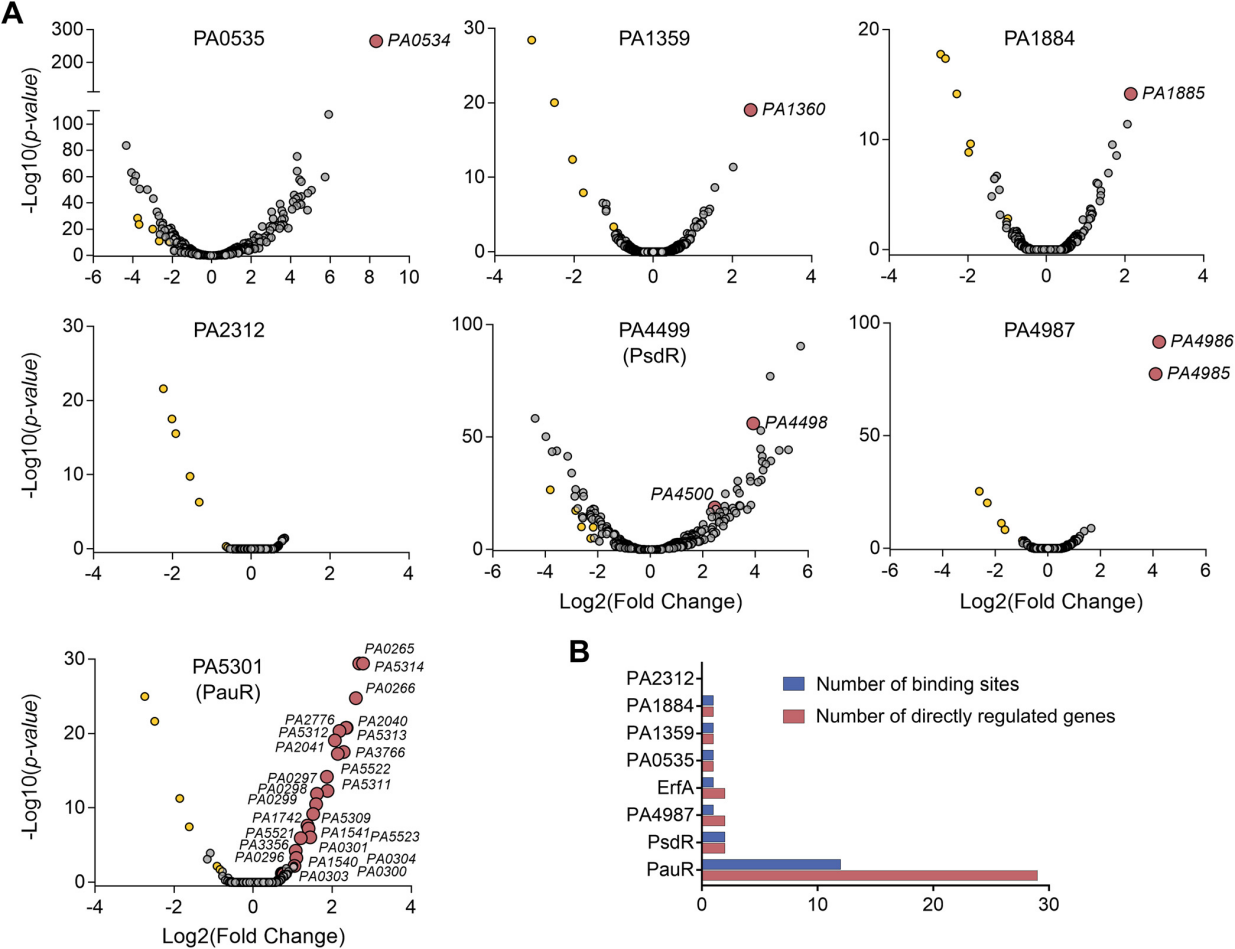
Determination of the regulons of the XRE-cupin regulators. (A) Volcano plots displaying the RNA-seq results of the genes differentially expressed in the respective XRE-cupin mutants versus parental strain PAO1. Genes for which a DAP-seq binding peak was identified in the promoter are represented by red circles and annotated with their gene ID. The genes (yellow circles) found commonly downregulated in all mutants represent artifacts probably due to genetic manipulation. (B) Summary of number of regulatory targets per TF in P. aeruginosa PAO1.

To get a global view on target specificities and regulatory networks of P. aeruginosa XRE-cupin TFs, we undertook to determine the regulons of all members of this family, except for ErfA, which we already comprehensively studied (8). To that aim, the corresponding genes were deleted in the genome of PAO1 and the transcriptomes of the engineered mutants were compared to that of the wild-type strain by RNA-seq. In parallel, we expressed and purified the seven recombinant regulators, and their targets were identified *in vitro* on fragmented P. aeruginosa PAO1 genome by DAP-seq (8, 15). Altogether, we determined transcriptomes and direct DNA targets for six of the seven regulators (Fig. 2A; see also Tables S3 and S4 in the supplemental material). The PA2312 protein was less soluble and stable than any of the other proteins and was thus probably inactive and/or aggregated, resulting in no significant peak detected in DAP-seq. In addition, the *PA2312* gene seems not to be expressed under the tested condition, as revealed by the few reads covering the coding DNA sequence (CDS) observed by RNA-seq in the wild-type strain. Overall, the family of XRE-cupin TFs regulates 39 genes in the PAO1 strain through direct binding to 19 genomic regions in total (Fig. 2B).

**FIG 3:**
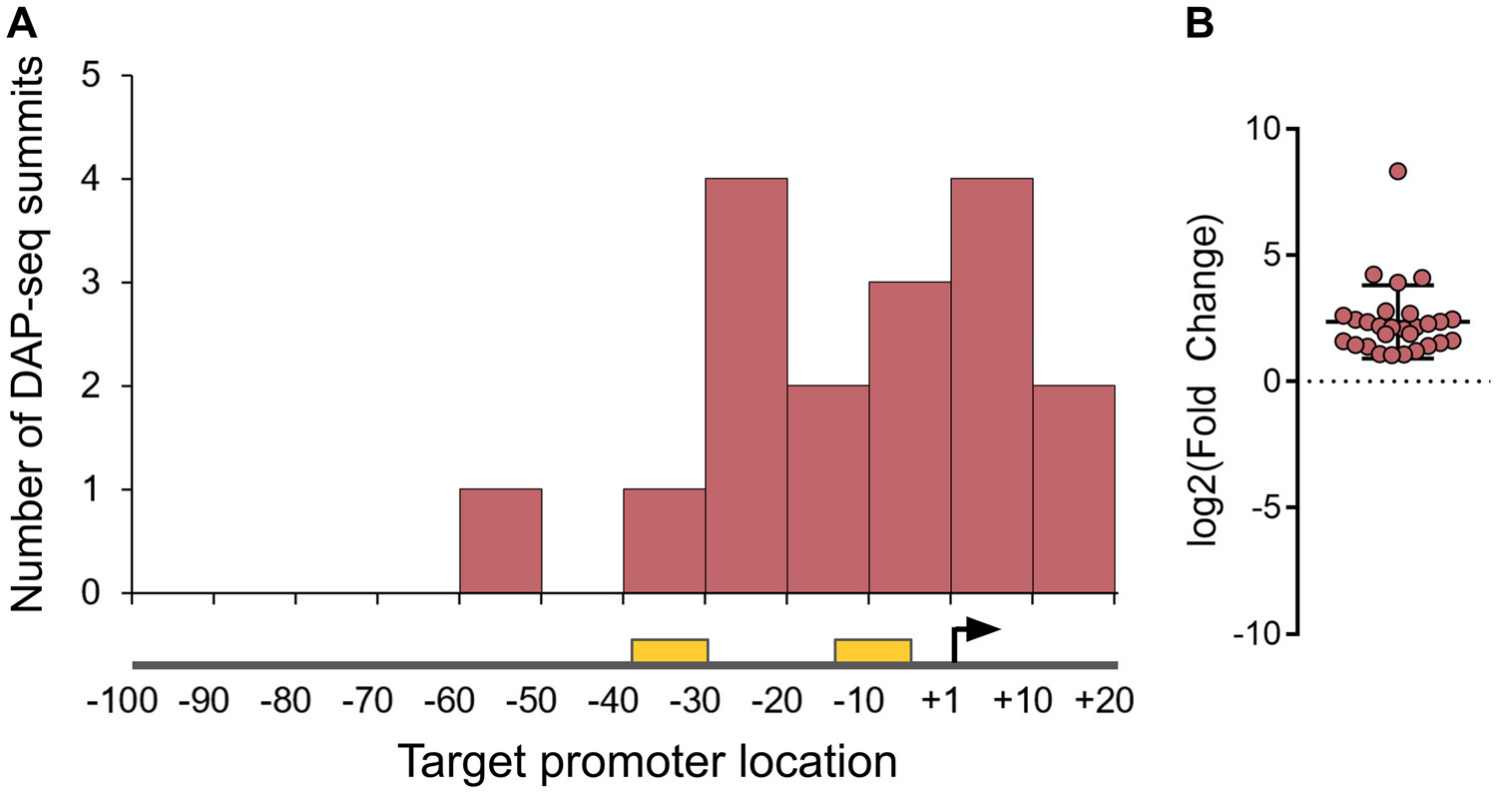
The XRE-cupin regulators are inhibitors of transcription. (A) Repartition of binding sites identified by DAP-seq within target promoters. RNA polymerase binding sites were either inferred from experimentally determined transcription start sites (44) or predicted using BPROM (45) if no data were available. The transcription start site is shown as a black arrow, and the −10 and −35 boxes are shown as yellow rectangles. (B) RNA-seq expression fold changes of target genes in regulator mutants compared to the wild-type strain.

**FIG 4:**
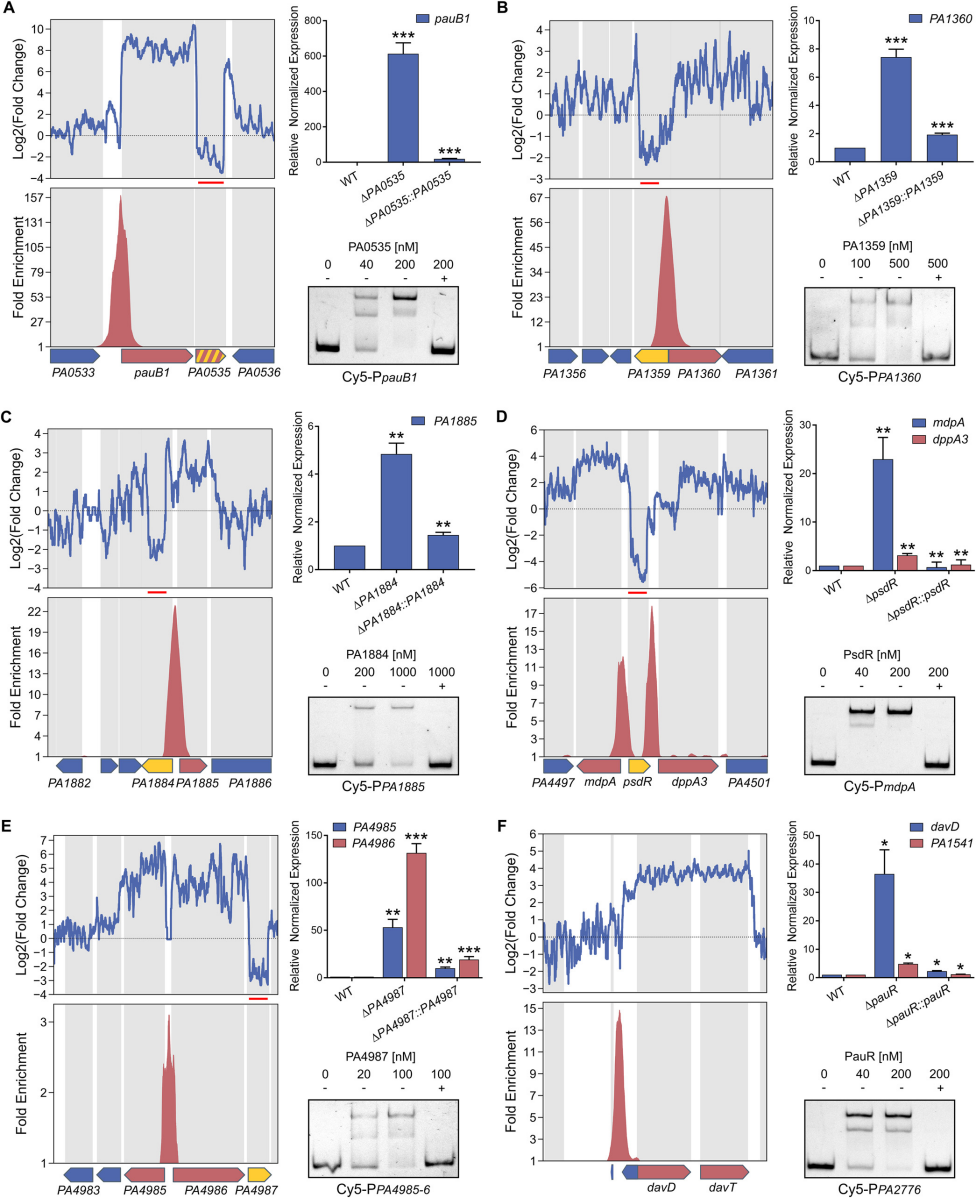
RT-qPCR and EMSA confirm the genome-wide results. Selected TFs and targets were PA0535 and *pauB1* (*PA0534*) (A), PA1359 and *PA1360* (B), PA1884 and *PA1885* (C), PsdR and *mdpA* (*PA4498*) and *dppA3* (*PA4450*) (D), PA4987 and *PA4985*-*PA4986* (E), and PauR and *davD* (*PA0265*), *PA1541* (by RT-qPCR), and *PA2776* (by EMSA) (F). (Upper left panels) Local RNA-seq read abundance fold changes in the corresponding mutants compared to parental strain. Red lines show the deleted region in the mutant strain. (Lower left panels) Local fold enrichments in the corresponding regulator obtained by DAP-seq compared to negative controls. Target genes are shown as red arrows, genes encoding the studied TF as yellow arrows, and others as blue arrows. Suspected autoregulation is denoted with dashed yellow-red arrows. (Upper right panels) RT-qPCR showing the regulation of the target genes in the corresponding regulatory mutants and complemented strains. Experiments were performed in triplicates and normalized to the *rpoD* transcripts. Error bars indicate the SD. Statistical significance was assessed using two-tailed *t* test (*P* value < 0.05 [*], 0.01 [**], or 0.001 [***]) and is shown against wild-type (WT) strains for mutants and against mutants for complemented strains. (Lower right panels) EMSA on target binding sites. Recombinant XRE-cupin-His_10_ proteins were incubated with 0.5 nM Cy5-labeled probes for 15 min before electrophoresis. For competition assays, excess of unlabeled probes (100 nM) is denoted by +.

In all cases, there was a strong correlation between *in vivo* and *in vitro* targets; the majority of DAP-seq peaks were centered on the promoters of genes that were also significantly dysregulated in RNA-seq. Most binding events occurred in the core promoter regions of regulated genes (Fig. 3A), and each target gene was found upregulated in the mutant of the corresponding regulator (Fig. 3B), showing that the XRE-cupin regulators are inhibitors of transcription. Our analyses confirmed the known regulon of PsdR and extended our knowledge on PauR, as discussed below. For most regulators, we found one or two direct targets, with the exception of PauR (PA5301), which directly regulates 29 genes (Fig. 2B). Some genes were found slightly downregulated in all mutants with no corresponding binding sites found, probably due to experimental conditions or genetic manipulations (Fig. 2A). At least one regulatory target per TF was further verified by RT-qPCR and electrophoretic mobility shift assays (EMSA), and in all cases the RNA-seq and DAP-seq results were confirmed by these targeted approaches (Fig. 4).

**FIG 5:**
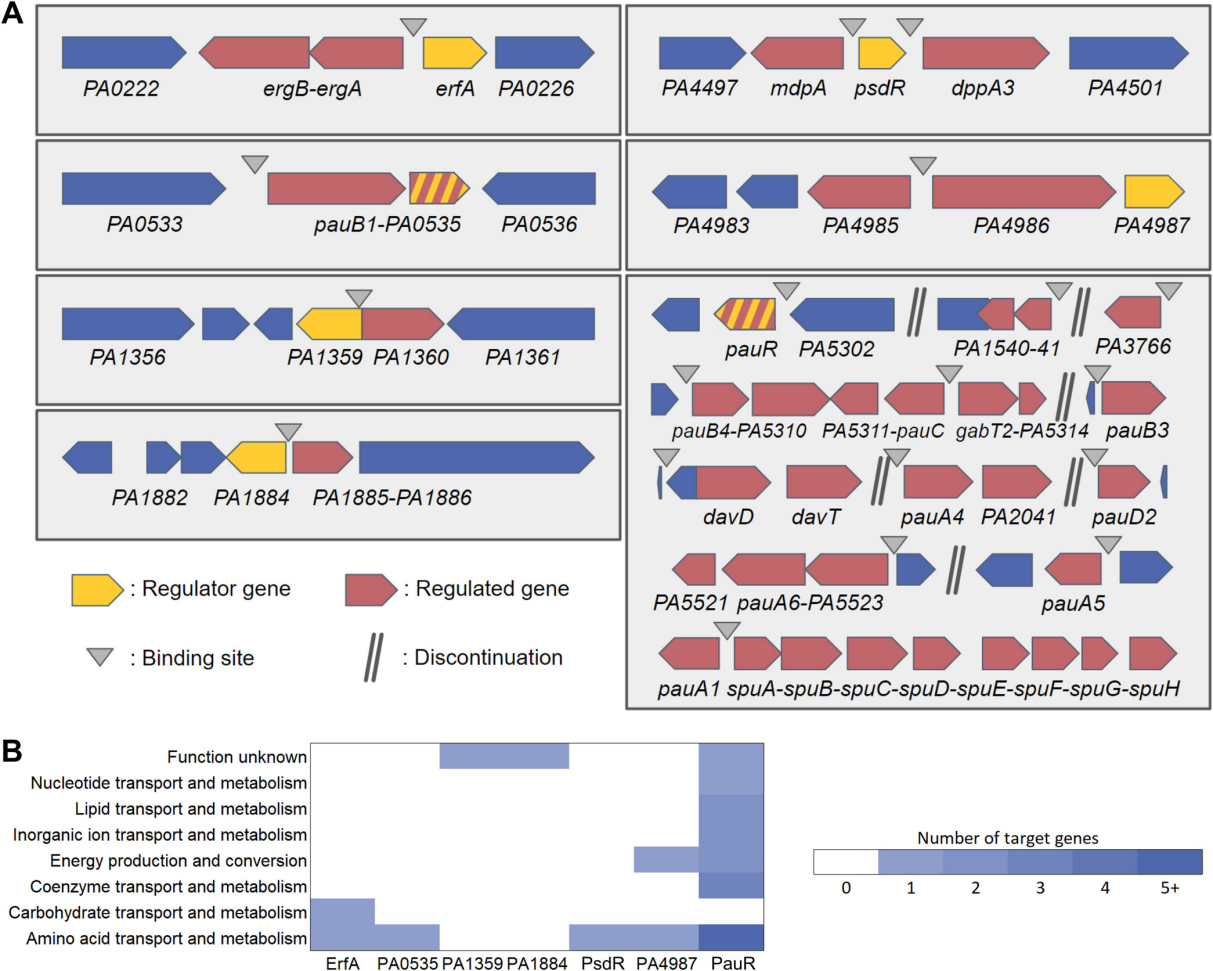
The complete regulons of the XRE-cupin regulators. (A) Schematic views of the local targeted regions of the seven XRE-cupin TFs with determined regulons in P. aeruginosa PAO1. Genes in operons, as annotated in the *Pseudomonas* genome database (18), are connected with hyphens. Genes that both have a DAP-seq peak in their promoter region and are differentially expressed (*P* value < 0.05) are shown as regulated genes (red arrows). (B) Functional annotation of XRE-cupin regulatory targets. COG functional annotations were retrieved from the PAO1 genome on the *Pseudomonas* database (18) for all target genes.

Strikingly, all the XRE-cupin TFs bind to at least one intergenic region directly adjacent to their own gene (Fig. 5). PauR strikes as an outlier with its 29 regulated genes scattered across 12 different genomic locations, while all other XRE-cupin repressors have one or two targets, always found in the direct vicinity of their own genes, showing that they are local, specialized regulators that form local functional units with their regulated genes. Horizontal gene transfer (HGT) is thought to shape the evolution of these gene groups and their regulatory relationships and might be at the origin of their differentiation from one another (16, 17).

Based on DAP-seq peaks, DNA-binding motifs could be generated for PsdR and PauR, which have more than one binding site (Fig. S1). These motifs exhibit a palindromic architecture, like the previously determined ErfA consensus (8), supporting DNA binding of XRE-cupin TFs as dimers. Mutations introduced into the binding site prevented PauR binding, further validating the inferred consensus (Fig. S1). Interestingly, mutation of only half the palindromic sequence recognized by PsdR led to a faster mobility fragment than that obtained with the wild-type sequence. This might correspond to the binding of one monomer to the mutated probe and supports the model in which the usual two-band shift observed with most XRE-cupin TFs corresponds to the binding of a monomer (lower, fainter band) and of a dimer (higher, stronger band) at higher concentration (Fig. 4 and Fig. S1). The three determined binding consensus sequences are quite different, probably due to differences in the amino acid composition of their DNA-binding interface observed among the TF family (Fig. S2). This supports their different DNA binding preferences and regulon specificities, as no common targets were revealed for any of the regulators.

10.1128/mSystems.00753-20.1 FIG S1 Validation of the DNA binding sites of PsdR and PauR. For PsdR (A) and PauR (B), from upper to lower panels: (i) enriched DNA motif obtained with MEME-ChIP in top DAP-seq peaks, (ii) local RNA-seq read abundance fold changes in the corresponding regulator mutants compared to the parental strain, (iii) local DAP-seq fold enrichments in the corresponding regulator experiments compared to negative controls, (iv) zoom view of the bound promoter region (mutated probes used in EMSA are shown with exchanged nucleotides in blue), and (v) EMSA of the corresponding region with either wild-type sequence or mutated probes. The mutation of half of the palindromic motif recognized by PsdR led to the shift of a lower band than with the wild-type probe, potentially representing the binding of only one monomer to the nonmutated half site. On the other hand, the mutation of the entire site for PauR led to a complete loss of binding. Download FIG S1, TIF file, 1.1 MB. Copyright © 2021 Trouillon et al. 2021 Trouillon et al. This content is distributed under the terms of the Creative Commons Attribution 4.0 International license.

10.1128/mSystems.00753-20.2 FIG S2 Predicted structures of DNA-binding interfaces of the eight XRE-cupin regulators. (A) Stereo ribbon representation of the predicted DNA-binding interfaces of the eight XRE-cupin regulators. Model prediction was done using the SWISS-MODEL tool (A. Waterhouse, M. Bertoni, S. Bienert, G. Studer, et al., Nucleic Acids Res 46:W296–W303, 2018, https://doi.org/10.1093/nar/gky427) and the structure with PDB ID 1Y9Q as the template. Amino acids predicted to be involved in specific DNA binding are depicted in yellow. (B) Sequence alignment of the eight XRE domains. The five alpha-helices are denoted by gray rectangles. Amino acids predicted to be involved in specific and nonspecific DNA binding are shown by red and gray arrows, respectively. Download FIG S2, TIF file, 1.5 MB. Copyright © 2021 Trouillon et al. 2021 Trouillon et al. This content is distributed under the terms of the Creative Commons Attribution 4.0 International license.

Altogether, we defined the regulons of seven TFs, completing our knowledge of the XRE-cupin TF family in P. aeruginosa (Fig. 5A). Our results show that all the members of the family are inhibitors of transcription, most likely acting by occluding the promoter and preventing RNA polymerase recruitment. Despite their common features (domain architecture, local action, inhibitory function), each XRE-cupin TF targets specific regulons without overlapping roles.

### XRE-cupin TFs regulate specific metabolic pathways.

To determine the functions of each TF, we scrutinized the roles of their regulons in detail (Fig. 5A and B). That of ErfA was already mentioned and encompasses the *ergAB* operon in PAO1, playing a putative role in amino acid or carbohydrate metabolism (8). PA0535 has only one target: it inhibits the *pauB1* gene and potentially its own expression, the two genes being predicted as an operon (18) (Fig. 4A and Fig. 5A). PauB1 is one of the four redundant PauB proteins, which are flavin adenine dinucleotide (FAD)-dependent oxidoreductases working in the complex γ-glutamylation pathway required for polyamine utilization in P. aeruginosa (14, 19). Although expression of its gene was shown to be increased in the presence of putrescine (20), PauB1 is required for cadaverine catabolism (14). We thus identified PA0535 as the missing regulator of the γ-glutamylation pathway, most of the genes being under the control of PauR, or BauR (19). Indeed, the key regulator PauR was shown to bind *in vitro* to eight sites and thus to potentially affect expression of 18 genes (14). As mentioned above, our results confirmed all known DNA targets of PauR, and RNA-seq provided further information concerning the extent of gene expression perturbation resulting from the TF binding. Two previously reported PauR binding sites were predicted to control the expression of six genes (14), while our genome-wide expression data show that they actually impact the expression of 13 genes, *pauA1* and the *spuABCDEFGH operon* for one, and the two divergently transcribed two-gene operons, *pauC*-*PA5311* and *gabT2*-*PA5314* for the other. Furthermore, we also extended the PauR regulon by identifying four additional binding sites, one located upstream of the *pauR* gene, strongly supporting an autoregulation mechanism. Three new identified targets were the *PA3766* gene encoding a probable amino acid/polyamine transporter and two operons, *davD-davT* and *PA1541-40*, which we confirmed by RT-qPCR (Fig. 4F). While *PA1541* encodes a probable transporter, the DavD and DavT proteins are enzymes involved in the conversion from 5-aminovalerate (AMV) to glutarate (19). Cadaverine is converted into AMV through the γ-glutamylation pathway, showing that PauR regulation extends beyond this metabolic pathway to further downstream steps. The PA1359 regulator negatively controls the expression of the *PA1360* gene (Fig. 4B), coding for a putative drug/metabolite transporter similar to the threonine exporter RhtA in Escherichia coli (21). PA1884 has also only one target, the *PA1885* gene (Fig. 4C), the product of which is a putative acyltransferase with a GNAT (Gcn5-related N-acetyltransferases) domain, which could either confer antibiotic resistance or have potential metabolic functions. Even if *PA1885* is predicted to be in an operon with the downstream *polB* gene (18), RNA-seq data indicated no difference on this gene, excluding a control by the inhibitor and suggesting the existence of a *polB*-specific promoter (Fig. 4C). The PsdR regulator was already identified as the repressor of *dppA3* and *mdpA* (12), involved in the uptake and utilization of small peptides; our results complete the scheme by demonstrating that the control is direct and involves two binding sites surrounding the *psdR* gene (Fig. 4D). DppA3 is a substrate-binding protein delivering tripeptides/dipeptides to the ABC transporter DppBCDF (22), and MdpA is a metallodipeptidase involved in their processing (12). Finally, PA4987 has one binding site in the intergenic sequence of *PA4985* and *PA4986*, downregulating expression of both genes as confirmed by RT-qPCR (Fig. 4E). Although *PA4984* is predicted to be in operon with *PA4985*, we did not observe any effect on its expression (Fig. 4E). PA4986 is a putative oxidoreductase, and PA4985 is a possible periplasmic spermidine/putrescine-binding protein. Overall, the XRE-cupin regulators seem to regulate functions related to specific amino acids or small-molecule uptake or processing (Fig. 5B).

Based on our knowledge of their different regulons, we attempted to further investigate the XRE-cupin TFs’ roles through two global phenotypic assays. We first took advantage of the Galleria mellonella infection model frequently used to assess overall bacterial fitness and virulence (23–25). No significant difference could be observed in survival curves between larvae infected with wild-type and mutant strains (Fig. S3). Therefore, the overexpression of genes regulated by the XRE-cupin family of repressors did not provide P. aeruginosa any advantage or disadvantage in this simple animal model. On the other hand, we assessed the strains’ antibiotic resistance to 24 different clinically relevant antibiotics and again found no differences (Fig. S3). These results reflect the specificity of the XRE-cupin regulators and their functional units toward precise conditions. Indeed, *psdR* inactivation was shown to increase growth fitness during proteolytic growth in caseinate medium, as this provides the dipeptide substrates for the derepressed dipeptides’ uptake and degradation (13). Also, *mdpA* expression is induced in the presence of X-Pro dipeptides that might explain the faster growth in their presence due to their higher metabolism (12). The fact that they do not affect fitness in global phenotypic assays illustrates that TFs of this family, along with their target genes, are niche-specific functional units allowing the bacteria to detect and respond to defined conditions.

10.1128/mSystems.00753-20.3 FIG S3 The XRE-cupin regulons do not affect bacterial fitness in G. mellonella, nor antibiotic resistance. (A) Survival curves of Galleria mellonella larvae infected with an average of 7 to 10 bacteria per larva. Twenty larvae were infected per strain. Significance testing was performed using log rank. (B) Antibiotic resistance phenotypes of the XRE-cupin regulatory mutants. Resistance to 24 clinically relevant antibiotics was assessed in liquid using the automated BD Phoenix system. Download FIG S3, TIF file, 0.7 MB. Copyright © 2021 Trouillon et al. 2021 Trouillon et al. This content is distributed under the terms of the Creative Commons Attribution 4.0 International license.

**FIG 6:**
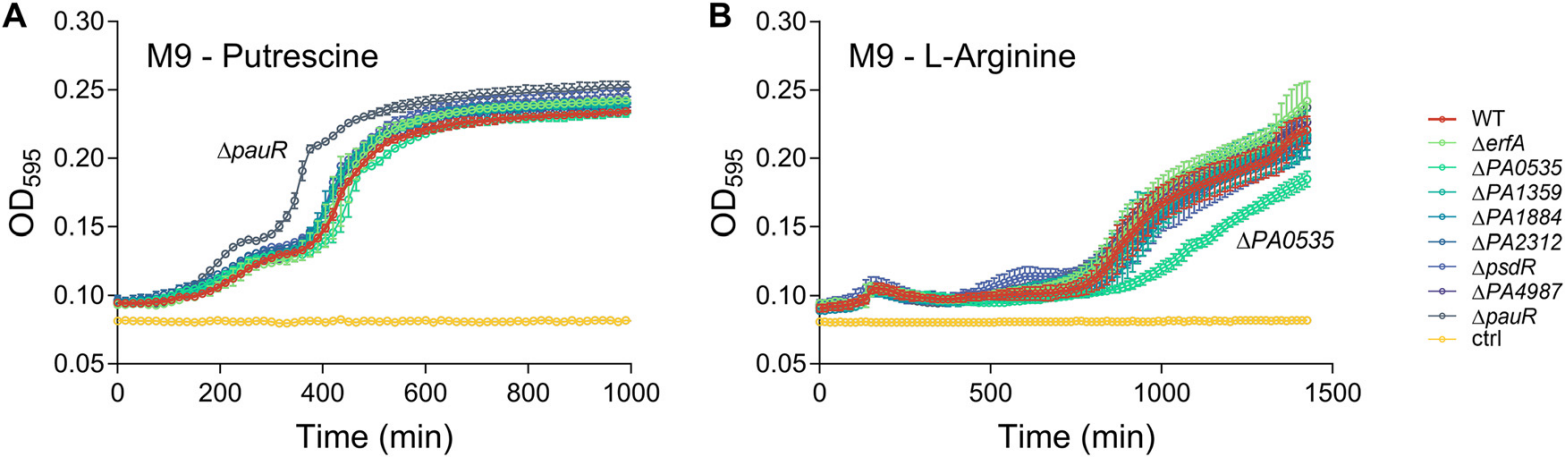
Growth of the strains deficient in XRE-cupin regulators on putrescine and arginine. Bacterial growth in M9 minimal medium containing putrescine (A) or l-arginine (B) as carbon source. After overnight growth at 37°C in LB, bacteria were diluted to an OD_600_ of 0.02 and grown in 96-well plates at 37°C with agitation for 24 h. Cultures were performed in biological duplicates.

To investigate the role of regulators under more specific conditions, in particular in relation to polyamines, we performed growth assays in LB medium and M9 minimal medium containing different carbon sources. While there were no differences in most growth media (Fig. S4), we identified a growth advantage of the *pauR* mutant when growing on putrescine and a growth defect of the *PA0535* mutant when growing on l-arginine (Fig. 6). It thus appears that disinhibition of the γ-glutamylation pathway due to the absence of PauR provides a growth advantage on putrescine, most probably due to the presence of all necessary enzymes for putrescine catabolism, allowing a faster growth initiation. Additionally, PA0535 inhibits the expression of *pauB1*, encoding one of four redundant PauB proteins involved in polyamine processing (20). Interestingly, l-arginine can be used as precursor for the biosynthesis of polyamines through the arginine decarboxylase (ADC) pathway (26), which suggests that PauB1 might play a role in the initiation of this pathway. Indeed, the overexpression of PauB1 in the PA0535 mutant could induce increased metabolic flux toward the ADC pathway and thus reduce l-arginine availability for more efficient catabolic pathways such as the arginine succinyltransferase (AST) pathway, thus explaining the growth defect of the PA0535 mutant. Altogether, these results provide new insights into the functions of XRE-cupin regulators and the complex γ-glutamylation pathway.

10.1128/mSystems.00753-20.4 FIG S4 Growth assays of the XRE-cupin mutant strains. Growth of PAO1 strains in LB or M9 minimal medium containing different carbon sources. After overnight growth at 37°C in LB, bacteria were diluted to an OD_600_ of 0.02 and grown in 96-well plates at 37°C with agitation for 24 h. Cultures were performed in biological duplicates. Download FIG S4, TIF file, 1.8 MB. Copyright © 2021 Trouillon et al. 2021 Trouillon et al. This content is distributed under the terms of the Creative Commons Attribution 4.0 International license.

It is hypothesized that XRE-cupin TFs are able to detect small signal molecules and detach from their DNA-binding sites upon sensing in order to induce expression of the target genes. PauR was indeed shown to be involved in the derepression of genes of the γ-glutamylation pathway in the presence of putrescine and cadaverine (14). In addition, the E. coli PauR homolog PuuR is known to dissociate from its binding site upon direct sensing of putrescine (27). In the same line, *mdpA* expression was shown to be induced in the presence of different dipeptides, probably sensed by PsdR (12). In all these cases, the signals were metabolites that are part or the precursors of the regulated metabolic pathways. The cupin domain in these regulators is predicted as a signal-sensing domain and thus could be responsible for the specific sensing of each regulon-related metabolite, as is often the case (28). The crystal structure of an XRE-cupin regulator from Vibrio cholerae has been solved and shows a binding pocket in the cupin domain with bound d-methionine (PDB ID 1Y9Q). The structure modelization of all eight P. aeruginosa XRE-cupin regulators also led to the identification of specific binding pockets in each cupin domain (Fig. S5). Similarly to what was found for their DNA-binding interfaces (Fig. S2), each regulator exhibits a different amino acid composition inside this putative signal-sensing pocket. These variabilities at the two major functional regions of the proteins explain the difference in DNA- and signal molecule-binding preferences and may result from evolutionary processes involving HGT that led to the multiplication and differentiation of these regulators.

10.1128/mSystems.00753-20.5 FIG S5 Structure modeling of the XRE-cupin putative signal-sensing binding pocket. Model prediction was done using the SWISS-MODEL tool and the structure with PDB ID 1Y9Q as the template. Inward amino acids potentially involved in molecule binding are drawn. Download FIG S5, TIF file, 2.2 MB. Copyright © 2021 Trouillon et al. 2021 Trouillon et al. This content is distributed under the terms of the Creative Commons Attribution 4.0 International license.

### XRE-cupin regulators are diverse and differently conserved between *Pseudomonas* species.

**FIG 7:**
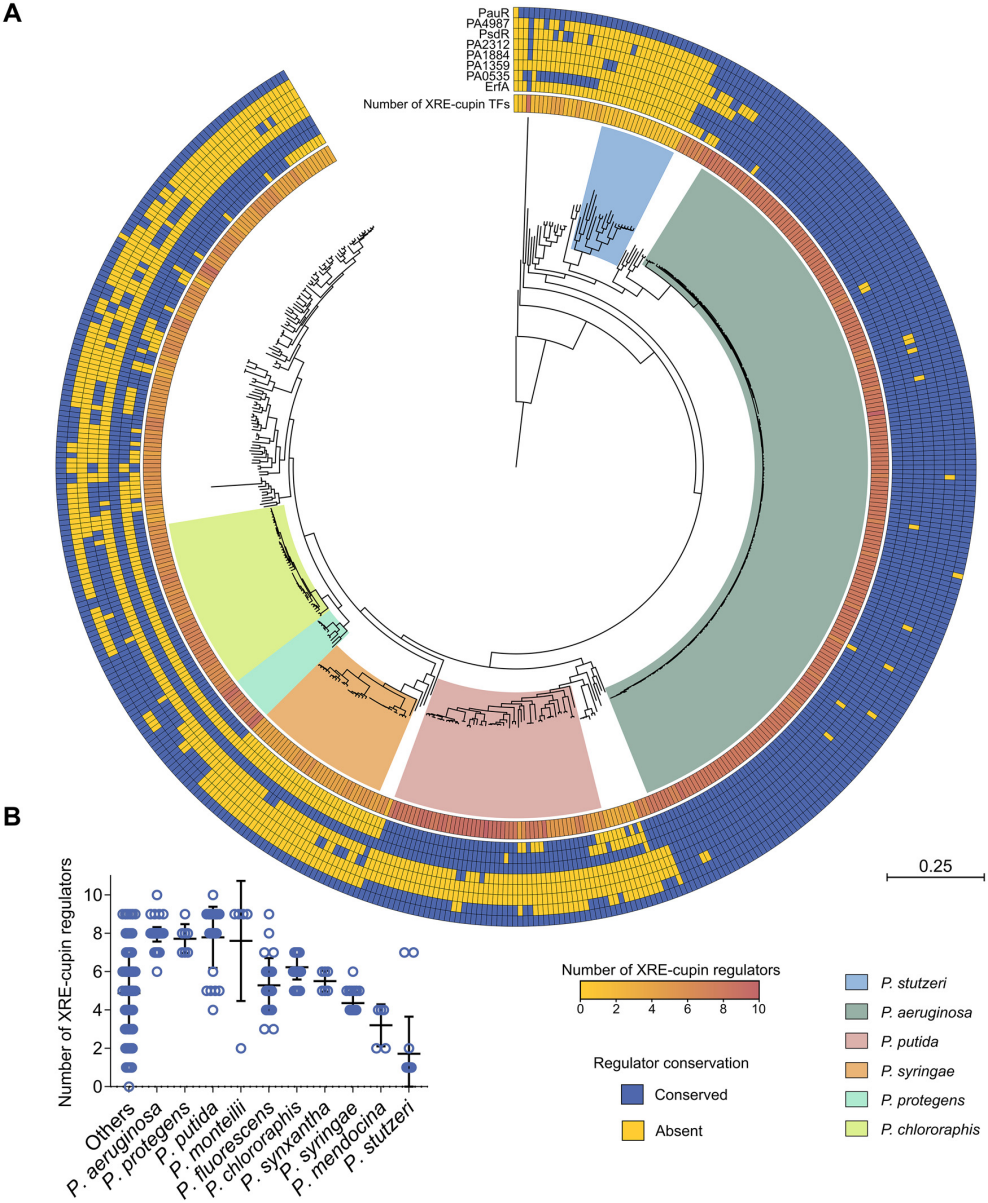
Phylogenetic analysis of the conservation of the XRE-cupin regulatory family across the *Pseudomonas* genus. (A) Maximum-likelihood phylogenetic tree of 503 *Pseudomonas* complete genomes. The tree was generated from the multiple alignment of the concatenated sequences of 66 core genes for each strain with 100 bootstraps. The most represented species are delineated with a colored background. The number of XRE-cupin proteins detected through the hidden Markov model search is shown as the inner circle yellow-to-red heatmap. The outer circles show the results of homolog search by Reciprocal Best BLAST Hit search for the eight regulators studied here. (B) Dot plot showing the distribution of number of XRE-cupin regulators per strain for each species. Species represented by fewer than five strains are grouped in the “Others” column.

As XRE-cupin TFs seem to regulate small individual metabolic pathways that could reflect the different environments encountered by the bacteria and their ability to adapt, we assessed the conservation of this family across the *Pseudomonas* genus. We first examined the conservation of the eight regulators studied here and found different conservation between *Pseudomonas* species (Fig. 7A). Interestingly, PA1359 and PauR were conserved in nearly all strains, suggesting a more central role or ancestral origin. The six other regulators are less conserved between strains and species. We can notice that although PA2312 seems not to be active in the PAO1 strain, its gene is conserved within P. aeruginosa strains and is present in other species, underlining its physiological importance. Here again, the conservation of the TFs and their regulatory targets probably depends on the environments encountered by each species which might require or not the associated metabolic functional units.

**FIG 8:**
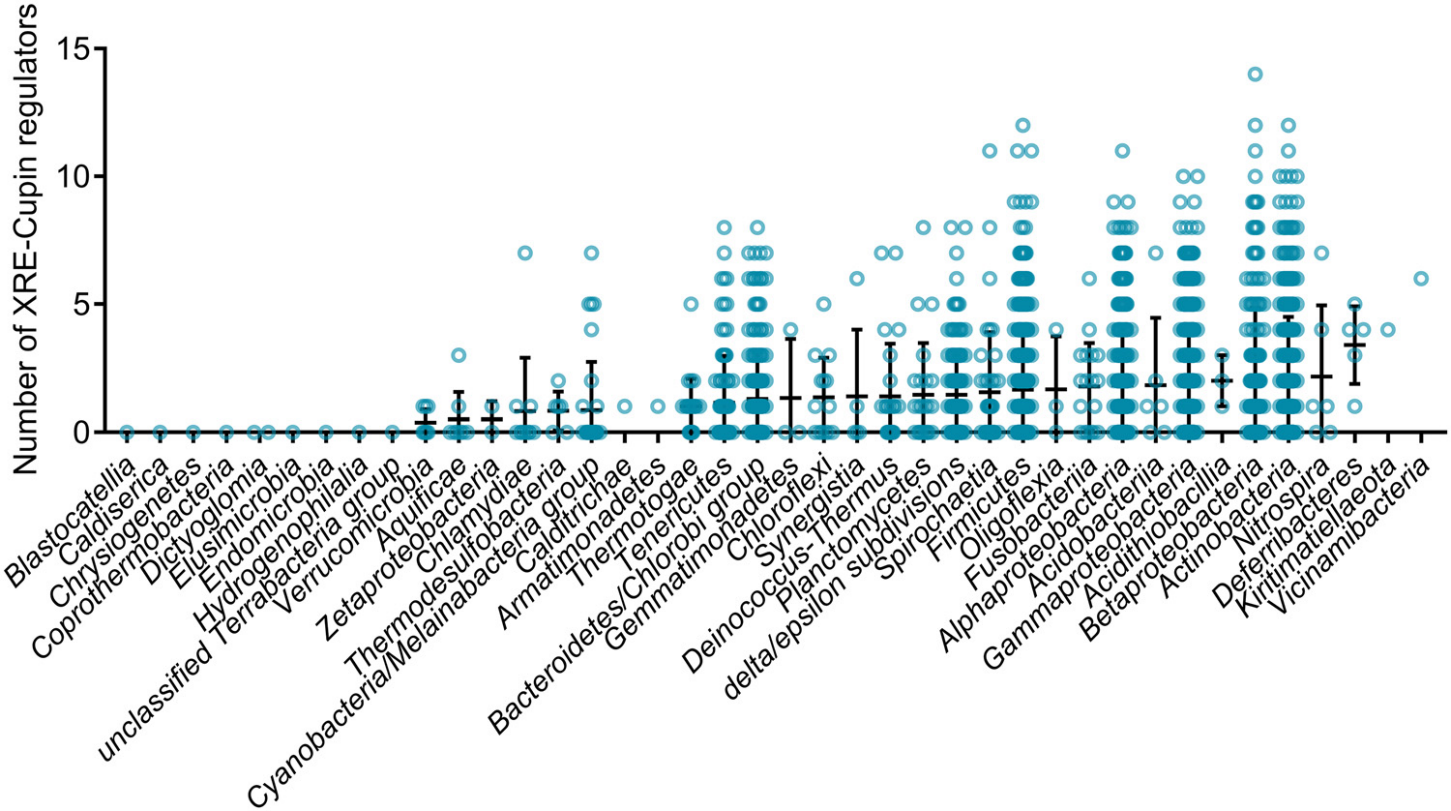
HMMER prediction of XRE-cupin regulators across the bacterial kingdom. Repartition of the number of predicted XRE-cupin regulators per genome. All 2,664 RefSeq representative complete bacterial genomes (>9.4 million unique protein sequences) were scanned using the XRE-cupin HMM and are shown in this figure across 40 different phylogenetic classes. Classes are sorted by increasing mean numbers of XRE-cupin regulators identified. A total of 4,515 XRE-cupin regulators were identified with HMM scores of >100 and were counted in this analysis.

To apprehend the importance of these regulators, we investigated the presence of other XRE-cupin TFs in all strains. Using all XRE-cupin sequences available on NCBI (*n *= 23,072) as the templates, we created and validated a hidden Markov model for the automated detection of such regulators. The model was able to specifically detect all XRE-cupin TFs in PAO1 (Fig. S6). Using this model, we screened nearly 3 million proteins retrieved from all *Pseudomonas* complete genomes for the presence of XRE-cupin regulators and identified 3,147 of them, with between zero and 10 present per strain, depending on the strain or species (Fig. 7A and B). Some species were found with only a few XRE-cupin TFs, fewer than two or three on average for some of them as, for example, in Pseudomonas mendocina or P. stutzeri. This could reflect the fact that these species are more niche specific and may not encounter a wide variety of environments and thus need fewer of these optional metabolic response functional units. On the other hand, species like P. aeruginosa, Pseudomonas protegens, or P. putida possess around eight and up to 10 XRE-cupin regulators. All three of these species are known for their versatility and capacity to adapt to a wide array of environmental conditions. The total number of XRE-cupin regulators per strain often encompasses TFs other than the eight studied here, showing that many more regulators of this family are present across the *Pseudomonas* genus, probably associated with as many or more different metabolic functional units. For instance, while only five of the eight TFs studied here are conserved in P. putida, most strains of this species possess eight or nine XRE-cupin TFs. Additionally, the search for XRE-cupin regulators across all representative complete bacterial genomes identified such regulators in 31 out of 40 tested phylogenetic classes (Fig. 8). Strains with relatively high numbers of XRE-cupin regulators, with up to 14 per strain, were found across *Proteobacteria*, *Firmicutes*, and *Actinobacteria*. Altogether, this exemplifies the diversity of this family of TFs and shows that many species or strains have evolved new functional units including new XRE-cupin regulators to respond to the specific environmental niches they might encounter.

10.1128/mSystems.00753-20.6 FIG S6 HMMER prediction of XRE-cupin regulators in *Pseudomonas*. (A) Repartition of HMMER scores of all 23,974 proteins found to match the XRE-cupin HMM. The bimodal distribution clearly delineates between false and true positives. All eight known P. aeruginosa XRE-cupin regulators had scores higher than 140, and all manually inspected predictions with scores of <100 were found not to be XRE-cupin proteins. Consequently, a threshold of >100 (denoted by a red line) was used to consider true XRE-cupin regulators. (B) Repartition of the number of predicted XRE-cupin regulators per strain. Download FIG S6, TIF file, 0.4 MB. Copyright © 2021 Trouillon et al. 2021 Trouillon et al. This content is distributed under the terms of the Creative Commons Attribution 4.0 International license.

### XRE-cupin regulators control neighboring enzyme-coding and metabolism-related genes.

**FIG 9:**
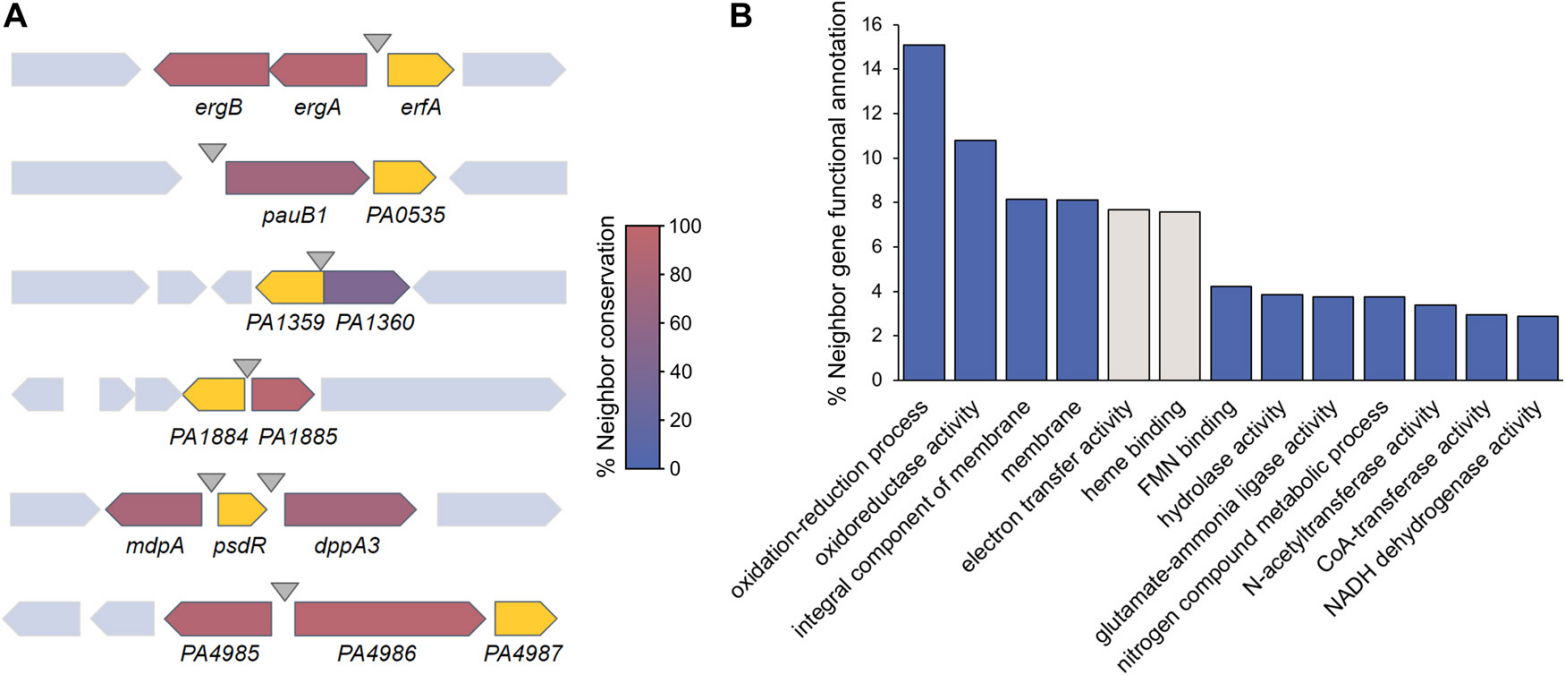
Gene synteny and genetic environment of the XRE-cupin regulators. (A) Conservation of XRE-cupin neighboring target genes. Regulator-encoding genes are shown in yellow. Target genes are colored depending on how often they were found as conserved neighbors of their associated XRE-cupin TF. (B) Histogram showing the proportion of the most represented (>2%) GO functional annotations. Functional annotations were obtained from Pfam results of InterProScan search on 6,294 genes neighboring XRE-cupin regulators from 503 *Pseudomonas* genomes. Two categories are shown in gray as they correspond to the *cycB* gene, a conserved neighbor of *pauR* not regulated by PauR, and thus are not representing XRE-cupin regulatory targets.

Six out of the seven XRE-cupin TFs experimentally characterized here regulate at least one gene adjacent to their own gene, forming local functional units. To investigate whether the local target regulation is universal for XRE-cupin TFs across the *Pseudomonas* genus, we first investigated the conservation of their neighboring genes (Fig. 9A). We found a high conservation of regulator/regulated gene pairs, highlighting the local nature of these functional units and the fact that they are exchanged by HGT as a group of genes between bacteria. To go further on the characterization of all XRE-cupin regulators, we assessed the functions of the neighbor genes of all 3,147 regulators identified in *Pseudomonas* here. Based on GO Term annotations of all 6,294 direct genetic neighbors, the vast majority of them encode either enzymes or small-molecule transporters (Fig. 9B). This result strongly corroborates the fact that local functional units comprising XRE-cupin regulators act as metabolic modules for the response to the presence of specific metabolites.

## DISCUSSION

In the present study, we characterized a family of eight TFs sharing the same domain architecture (XRE-cupin TFs) in P. aeruginosa and identified their regulatory targets. The XRE-cupin family members are exclusive inhibitors of the transcription of metabolism-related genes probably needed under defined, specific conditions. The current model is that these TFs act as regulatory switches that keep the transcription of their neighboring target genes down until their products are needed for a given metabolic pathway. Upon sensing of the precursor metabolite of the regulated pathway through their cupin domain, they disengage from the DNA in order to allow transcription of the genes coding for enzymes or transporters that will process the sensed molecule. This could include catabolism for nutrient source, as seen for PsdR, or detoxification via export of sensed molecules, as predicted for PA1359.

In most cases, the target genes were found located in the direct vicinity of the regulator gene, revealing the local nature of these regulatory interactions. Neighbor regulation is a common feature in bacteria, which has been explained by different models (16, 29). Genes that are functionally related, including regulators and regulated targets, tend to regroup together on the chromosome in order to be a complete functional unit when transferred by HGT. Different types of neighbor regulation are known (16), three of which were found in this study: simple neighbor regulation and coregulation of neighbors in both *cis* and *trans*, with some instances of autoregulation. In all cases, the total number of targets was small, allowing the formation of these local functional units. The very specific functions of the XRE-cupin regulons explain the small number of regulated genes and the fact that these units are local and probably easily transferrable gene clusters.

We found a high conservation of some of the studied TFs and their neighboring target genes across the *Pseudomonas* genus. These functional units may have been exchanged by HGT between bacteria that encounter similar environmental conditions. Additionally, a large number of different XRE-cupin regulators, each associated with their specific genetic neighborhood, were identified across the *Pseudomonas* genus. Out of the eight TFs studied here, none shared any common regulatory targets, and the two known sensed signals (dipeptides and polyamines) are different. This is explained by differences found at both DNA-binding and signal-sensing interfaces. Such differences are thought to happen through evolution, after and during repurposing or duplication events, and explain the presence of different XRE-cupin regulators in each species. The large number of this type of regulator illustrates their ancestral nature and their importance as condition-specific, transferrable means of metabolic diversification.

*Pseudomonas* species are famous for their wide metabolic versatility, encompassing many condition-specific metabolic pathways (30). While the prediction of metabolism-related functions works relatively well, experimental studies are still needed to pinpoint the exact pathways represented by uncharacterized genes. As illustrated by the target genes found here which mostly have only predicted functions, there is still a great need of phenotypic characterization of putative new metabolic pathways. Such studies would complete our knowledge on the mode of action of the XRE-cupin family of regulators and help understand both the function and sensed signals of this class of TFs. We also believe that family-wide and interspecies studies of TFs should become more common in order to be able to decipher global regulatory networks, especially in bacteria where they are still scarce.

## MATERIALS AND METHODS

### Bacterial strains.

The bacterial strains used in this study are listed in Table S1 in the supplemental material. P. aeruginosa and E. coli strains were grown in lysogeny broth (LB) at 37°C under agitation (300 rpm). P. aeruginosa strains were selected on *Pseudomonas* isolation agar (PIA) plates. Antibiotics were added when needed at the following concentrations: for P. aeruginosa, 200 μg/ml carbenicillin, 200 μg/ml gentamicin, and 200 μg/ml tetracycline; for E. coli, 100 μg/ml ampicillin, 50 μg/ml gentamicin, and 10 μg/ml tetracycline.

10.1128/mSystems.00753-20.7 TABLE S1 List of bacterial strains and plasmids used in this study. Download Table S1, PDF file, 0.6 MB. Copyright © 2021 Trouillon et al. 2021 Trouillon et al. This content is distributed under the terms of the Creative Commons Attribution 4.0 International license.

For growth assays, after overnight growth at 37°C in LB, bacteria were diluted to an optical density at 600 nm (OD_600_) of 0.02 in 200 μl of LB or M9 minimal medium (48 mM Na_2_HPO_4_, 22 mM KH_2_PO_4_, 9 mM NaCl, 19 mM NH_4_Cl) supplemented with 2 mM MgSO_4_, 0.1 mM CaCl_2_, and different carbon sources with equal concentrations of carbon ([C] = 66 mM): 0.2% glucose, 0.24% pyruvate, 0.28% acetate, 0.2% Casamino Acids, 0.2% succinate, 0.27% putrescine, or 0.23% l-arginine. Growth in 96-well plates was assessed by following the OD_595_ using a microplate reader for 24 h at 37°C with agitation.

### Plasmids and genetic manipulations.

Plasmids and primers are listed in Tables S1 and S2, respectively. For overproduction of recombinant His_10_-tagged proteins, each gene sequence was amplified by PCR using PAO1 genomic DNA as a matrix and appropriate primer pairs and then integrated by sequence- and ligation-independent cloning (SLIC) in pET-52b cut with NcoI-SacI and sequenced.

10.1128/mSystems.00753-20.8 TABLE S2 Primers used in this work. Download Table S2, PDF file, 0.5 MB. Copyright © 2021 Trouillon et al. 2021 Trouillon et al. This content is distributed under the terms of the Creative Commons Attribution 4.0 International license.

10.1128/mSystems.00753-20.9 TABLE S3 RNA-seq results. Download Table S3, XLSX file, 3.6 MB. Copyright © 2021 Trouillon et al. 2021 Trouillon et al. This content is distributed under the terms of the Creative Commons Attribution 4.0 International license.

10.1128/mSystems.00753-20.10 TABLE S4 DAP-seq results. Download Table S4, XLSX file, 0.1 MB. Copyright © 2021 Trouillon et al. 2021 Trouillon et al. This content is distributed under the terms of the Creative Commons Attribution 4.0 International license.

To generate P. aeruginosa deletion mutants, upstream and downstream flanking regions of each gene were amplified using appropriate primer pairs (sF1/sR1 and sF2/sR2 for each construct). The two resulting, overlapping fragments were then cloned into SmaI-cut pEXG2 by SLIC (31) and sequenced.

For complementation of *PA1359*, *PA1884*, *PA4499*, and *PA5301* mutants, a fragment encompassing the promoter (around 500 bp upstream from the start codon) and the CDS was amplified by PCR and cloned by SLIC into the mini-CTX1 plasmid cut by SmaI. When the gene was predicted as the second gene of an operon (*PA0535*, *PA2312*, and *PA4987*) (18), the operon promoter was directly fused to the regulator gene. To do so, two fragments were generated, the upstream fragment carrying around 500 bp upstream and the first 3 codons of the first gene of the operon and the second one carrying the last few codons and the entire CDS of the gene of interest. Then, the two overlapping fragments were cloned by SLIC in pEXG2 and sequenced.

All mini-CTX1- and pEXG2-derived plasmids were transferred by triparental mating into P. aeruginosa, using the pRK600 helper plasmid. For allelic exchange, merodiploids resulting from cointegration events were selected on PIA plates containing gentamicin. Colonies were then plated on NaCl-free LB agar plates containing 10% sucrose to select for the loss of plasmid. The resulting sucrose-resistant strains were checked for gentamicin sensitivity, and the mutant genotypes were determined by PCR. For complementation using mini-CTX1-derived plasmids, bacteria with an *att* site-inserted plasmid were selected on PIA plates containing tetracycline, and then they were cured from the mini-CTX1 backbone by excising the FLP recombination target (FRT) cassette with pFLP2 plasmid as previously described (32).

### Structure modeling.

Protein sequences from the eight P. aeruginosa XRE-cupin TFs were aligned against the RCSB Protein Data Bank (33) in order to find an experimentally obtained three-dimensional (3D) structure to use as a template for modeling. The 1.90-Å 3D structure of an XRE-cupin transcription factor from V. cholerae (PDB ID 1Y9Q) was used as the template as it permitted the best modeling confidence using the SWISS-MODEL tool (34).

### Protein purification.

All the pET-52b plasmids were transformed into E. coli BL21 Star(DE3). For protein overproduction, overnight cultures were diluted to an OD_600_ of 0.05 in LB medium containing 100 μg/ml ampicillin, and expression was induced at an OD_600_ of 0.6 with 1 mM IPTG. After 3 h of growth at 37°C, bacteria were harvested by centrifugation at 6,000 × *g* for 10 min at 4°C and resuspended in L buffer (25 mM Tris-HCl, 500 mM NaCl, 10 mM imidazole, 1 mM phenylmethylsulfonyl fluoride [PMSF], 5% glycerol, pH 8, containing Roche protease inhibitor cocktail). Bacteria were then lysed by sonication. After centrifugation at 66,500 × *g* for 30 min at 4°C, the soluble fraction was directly loaded onto a 1-ml nickel column (Protino Ni-nitrilotriacetic acid [NTA]; Macherey-Nagel). The column was washed with wash buffer (50 mM Tris-HCl, 500 mM NaCl, 5% glycerol, pH 8) containing increasing imidazole concentrations (20, 40, and 60 mM), and proteins were eluted with 200 mM imidazole. Aliquots from the peak protein fractions were analyzed by SDS-PAGE, and the fractions containing the proteins of interest were pooled and dialyzed against ErfA buffer (50 mM Tris-HCl, 250 mM NaCl, 50 mM KCl, 10% glycerol, 0.5% Tween20, pH 7).

### DAP-seq experimental procedure, sequencing, and data analysis.

DAP-seq was carried out in triplicates on PAO1 genomic DNA exactly as previously described (8). Sequencing was performed by the high-throughput sequencing core facility of I2BC (http://www.i2bc.paris-saclay.fr) using an Illumina NextSeq500 instrument. Approximately 8 million single-end reads per sample were generated on average with >90% of reads uniquely aligning to the PAO1 genome. Data analysis was performed as previously described (8). Briefly, peaks were generated with MACS2 (35) for each replicate against a pool of the three control samples (with *q* value of <0.05), and peaks present in all three replicates were selected for further analysis.

### RNA isolation.

P. aeruginosa strains were grown from overnight cultures diluted to an OD_600_ of 0.1 in 3 ml of fresh LB medium at 37°C under agitation in duplicate. Total RNA was isolated at an OD_600_ of 1.0 using hot phenol-chloroform extraction as previously described (8).

### RNA-seq library construction, sequencing, and data analysis.

After RNA isolation and DNase treatment, RNA sample quality was assessed on an Agilent Bioanalyzer, yielding RNA integrity numbers (RINs) of 9 or higher. Then, rRNAs were depleted using the RiboMinus transcriptome isolation kit (ThermoFisher) following manufacturer instructions. The cDNA libraries were then constructed from 50 ng of depleted RNA using the NEBNext Ultra II directional RNA library prep kit following manufacturer instructions (New England Biolabs [NEB]). Libraries were size-selected to 200 to 700 bp using SPRIselect beads, and quality was assessed on the Agilent Bioanalyzer using high-sensitivity DNA chips. Sequencing was done on an Illumina NextSeq500, and approximately 12 million single-end reads per sample were generated. Data analysis was performed as previously described (8).

### RT-qPCR.

After total RNA isolation and DNase treatment, cDNA synthesis was carried out using 2 μg of RNA as previously described (8). The experiments were performed with 3 biological replicates for each strain, and the relative expression of mRNAs was analyzed with the CFX Manager software (Bio-Rad) using the Pfaffl method relative to *rpoD* reference quantification cycle (*C_q_*) values. Statistical analyses were performed by two-tailed *t* test. The sequences of primers are listed in Table S2 in the supplemental material.

### Electrophoretic mobility shift assay.

Genomic DNA was used as a matrix to generate wild-type sequence probes. To amplify probes bearing mutagenized binding sites, three different matrices were created: the one with a mutated binding site in P*_PA0534_* resulted from the fusion of two overlapping fragments generated by primer pairs pEXG2-mut-PA0534-BS-sF1/sR1 and pEXG2-mut-PA0534-BS-sF2/sR2. PCR fragments with modified binding sites in P*_PA2776_* and P*_mdpA_* were produced by using primer pairs CTX-PA2776-*lacZ*-sF/PA2776-mut-*lacZ*-sR and CTX-mdpA*-lacZ*-sF/mdAmut-*lacZ* sR, respectively. Then, all fluorescent DNA probes were generated by PCR in two steps: a first PCR using specific primer pairs amplified the target DNA regions (83 to 96 bp) flanked by a 21-bp region which is targeted in a second PCR by a single Cy5-labeled primer. The resulting labeled probes were purified on DNA cleanup columns (NEB) and then incubated at 0.5 nM for 5 min at 37°C in EMSA buffer (10 mM Tris-HCl, 50 mM KCl, 10 mM MgCl_2_, 10% glycerol, 0.1 mg/ml bovine serum albumin [BSA], pH 8) containing 25 ng/μl poly(dI-dC). For competition assays, 100 nM unlabeled DNA probes (200-fold excess) were incubated with the labeled probes. Recombinant proteins were added at the indicated concentration in a final reaction volume of 20 μl and incubated for an additional 15 min at 25°C. Samples were then loaded on a native 8% Tris-acetate-EDTA (TAE) polyacrylamide gel and run at 100 V and 4°C in cold 1× TAE buffer. Fluorescence imaging was performed using a ChemiDoc MP.

### Galleria mellonella infection assay.

G. mellonella larva infections were performed as previously described (8). Animals injected with sterile phosphate-buffered saline (PBS) served as a control for physical trauma. CFU for each dilution were systematically counted from the insulin pen and were 7 ± 1 CFU per injection. Twenty larvae were injected per condition. Infection development was followed for 24 h at 37°C, and the animals were considered dead when not reacting to touch. Strains were independently randomized and blinded, ensuring no bias in CFU spotting and counting, and in animal death counting, which was also done by a different person. Statistical significance was assessed using a log rank test.

### Antibiotic resistance screening.

Antimicrobial susceptibility was assessed by broth microdilution using the automated BD Phoenix system (Becton, Dickinson, France) according to the manufacturers’ recommendations. In brief, colonies were suspended in BD Phoenix ID broth to obtain approximately a 0.5 McFarland (1.5 × 10^8^-CFU/ml) suspension. Broth was then placed on a BD Phoenix AP system that automatically adjusts the optical density and further dilutes the bacterial suspension in BD Phoenix AST broth (a cation-adjusted formulation of Mueller-Hinton broth containing 0.01% Tween 80) to obtain a final bacterial concentration in the AST broth of approximately 5 × 10^5^ CFU/ml. A redox indicator was then added to the AST broth. Inoculated AST broth was then poured into a BD Phoenix NMIC-417 panel, loaded into the BD Phoenix M50 instrument, and incubated at 35°C ± 1°C. Results were interpreted by the BD EpiCenter system according to CASFM-EUCAST (European Committee on Antimicrobial Susceptibility Testing) 2019 V1 breakpoints.

### Phylogenetic analysis and XRE-cupin identification.

All *Pseudomonas* complete genomes (*n *= 503) were retrieved from the *Pseudomonas* Genome Database (18). The sequences from 66 core genes were concatenated for each genome, and a multiple alignment was performed with MAFFT Galaxy version 7.221.3 (36) using default settings. The resulting alignment was used to build a maximum-likelihood phylogenetic tree using MEGA X with 100 bootstraps which was visualized and annotated using iTOL v5 (37, 38). XRE-cupin homolog identification was performed by Reciprocal Best BLAST Hit (RBBH) analysis (39) on the European Galaxy server (40) using protein sequences from P. aeruginosa PAO1 against all protein sequences from the other 502 genomes.

For XRE-cupin identification, the ErfA protein sequence was used to retrieve all protein sequences (*n *= 23,072) with an XRE-cupin domain architecture (HTH_3 and Cupin_2) from the NCBI Conserved Domain Architecture Retrieval Tool (41). A multiple alignment was performed on these sequences using MAFFT Galaxy version 7.221.3 (36) with default settings. The resulting alignment was used to build a hidden Markov model (HMM) using hmmbuild from HMMER (42). All 2,862,589 protein sequences from the 503 *Pseudomonas* genomes were then scanned through the model using hmmscan from HMMER (42). A bimodal distribution of HMMER scores could be observed, which led to the determination of a true-positive XRE-cupin threshold (HMMER score >100), which was validated by functional prediction from both sides (Fig. S6). All proteins above this threshold (*n *= 3,147) were considered XRE-cupin regulators.

For XRE-cupin identification across phylogenetic classes, all RefSeq representative complete bacterial genomes were retrieved from NCBI (*n = *2,664) and their protein sequences (>9.4 million unique sequences) were scanned through the XRE-cupin model using hmmscan from HMMER (42).

### Genetic neighbor functional prediction.

Gene IDs from all proteins matching the HMM were used to identify the direct upstream and downstream neighbor genes from all 503 genome annotation files using Python 3.7. The corresponding 6,294 protein sequences were then used for functional prediction analysis using the InterProScan functional prediction tool (43). GO annotations associated with Pfam predicted functions were then used for functional prediction.

### Data availability.

RNA-seq and DAP-seq data files have been deposited in National Center for Biotechnology Information's Gene Expression Omnibus (GEO) and can be accessed through GEO Series accession numbers GSE155521 and GSE155522, respectively.
