# Supplementary figures and images for: Transcription Inhibitors with XRE DNA-Binding and Cupin Signal-Sensing Domains Drive Metabolic Diversification in Pseudomonas
Source: mSystems. 2021 Jan 12;6(1):e00753-20. doi: 10.1128/mSystems.00753-20 (PMC7901475; doi:10.1128/mSystems.00753-20)

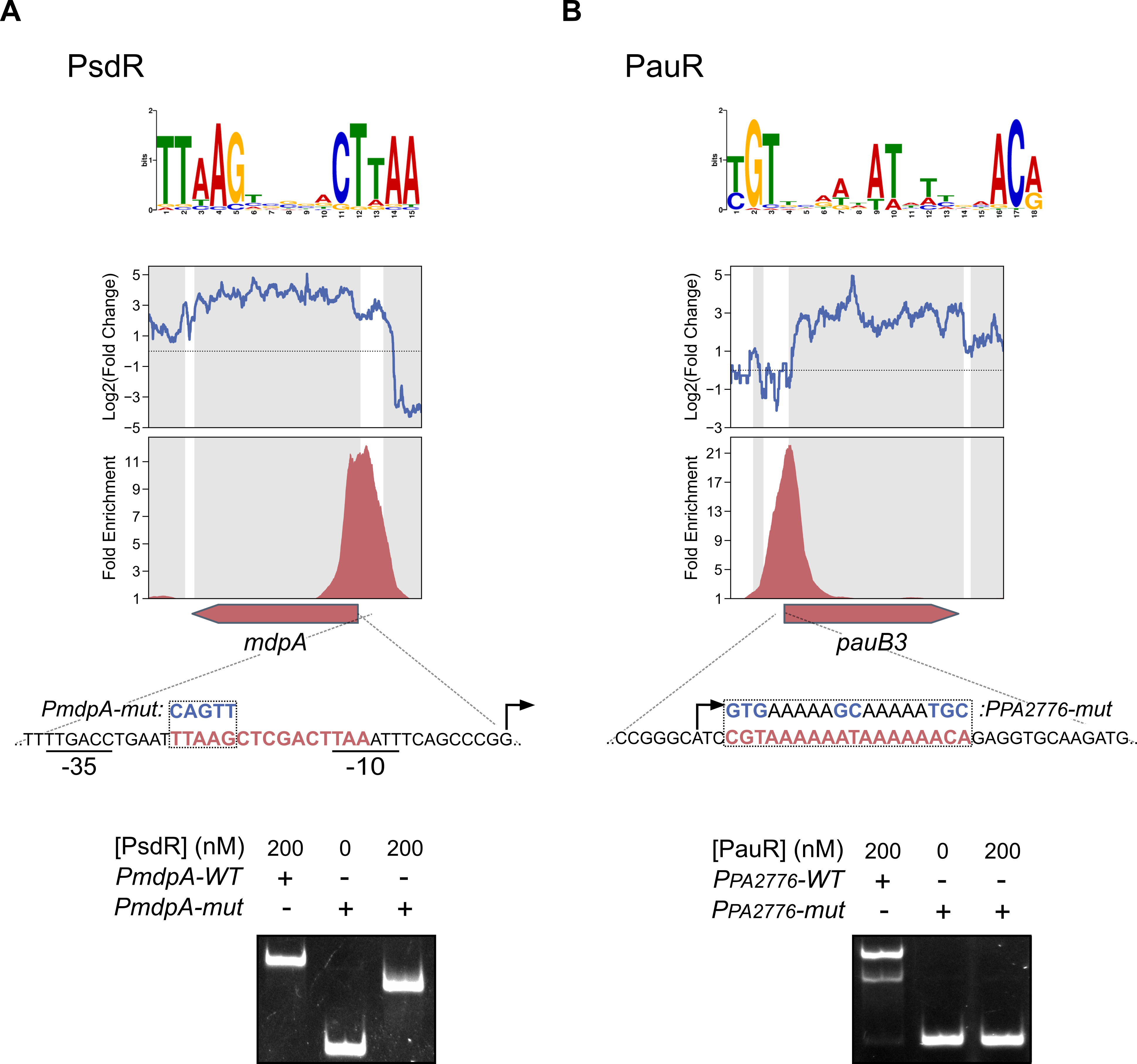

Supplement: FIG S1 [file mSystems.00753-20-sf001.tif]

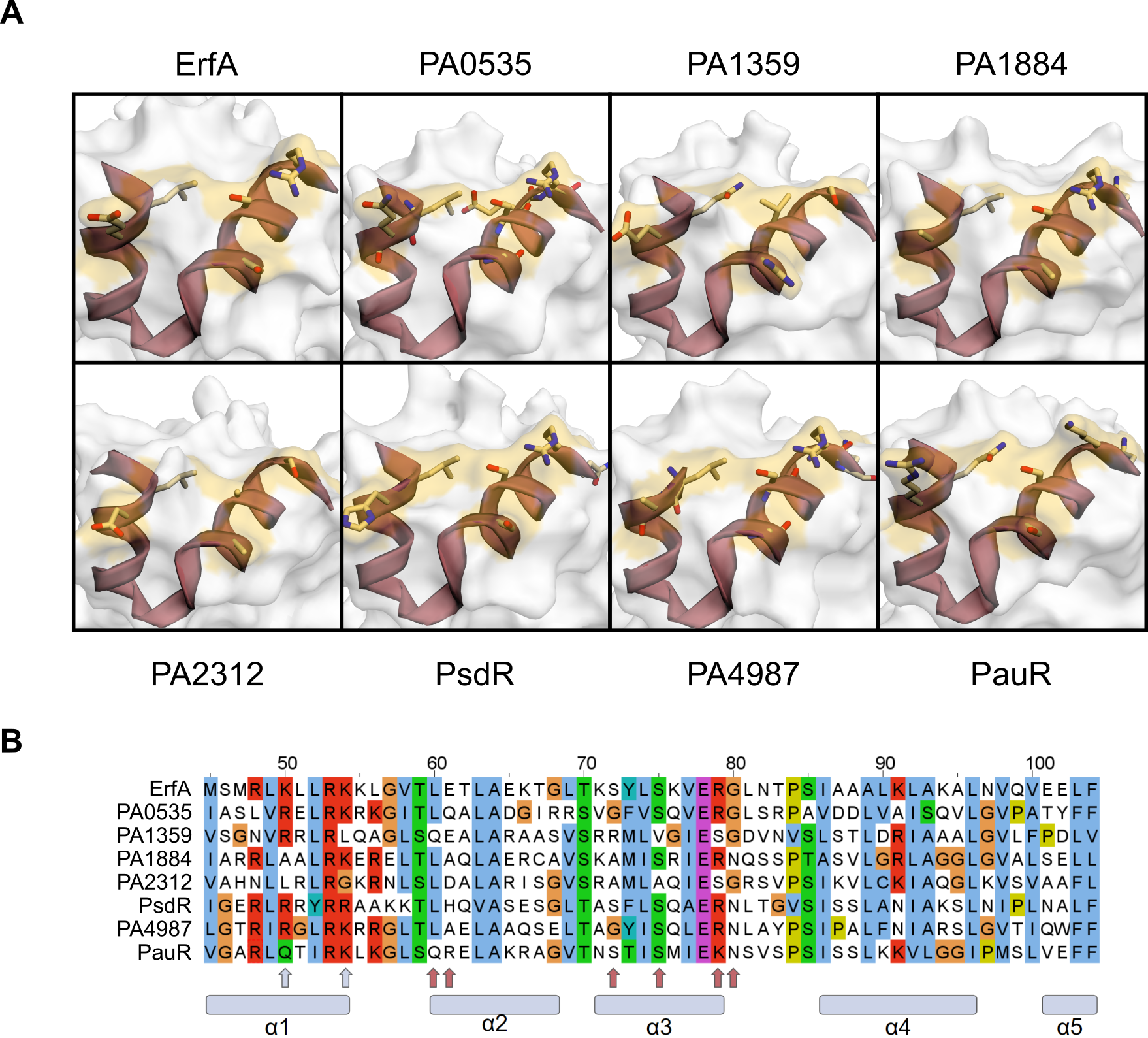

Supplement: FIG S2 [file mSystems.00753-20-sf002.tif]

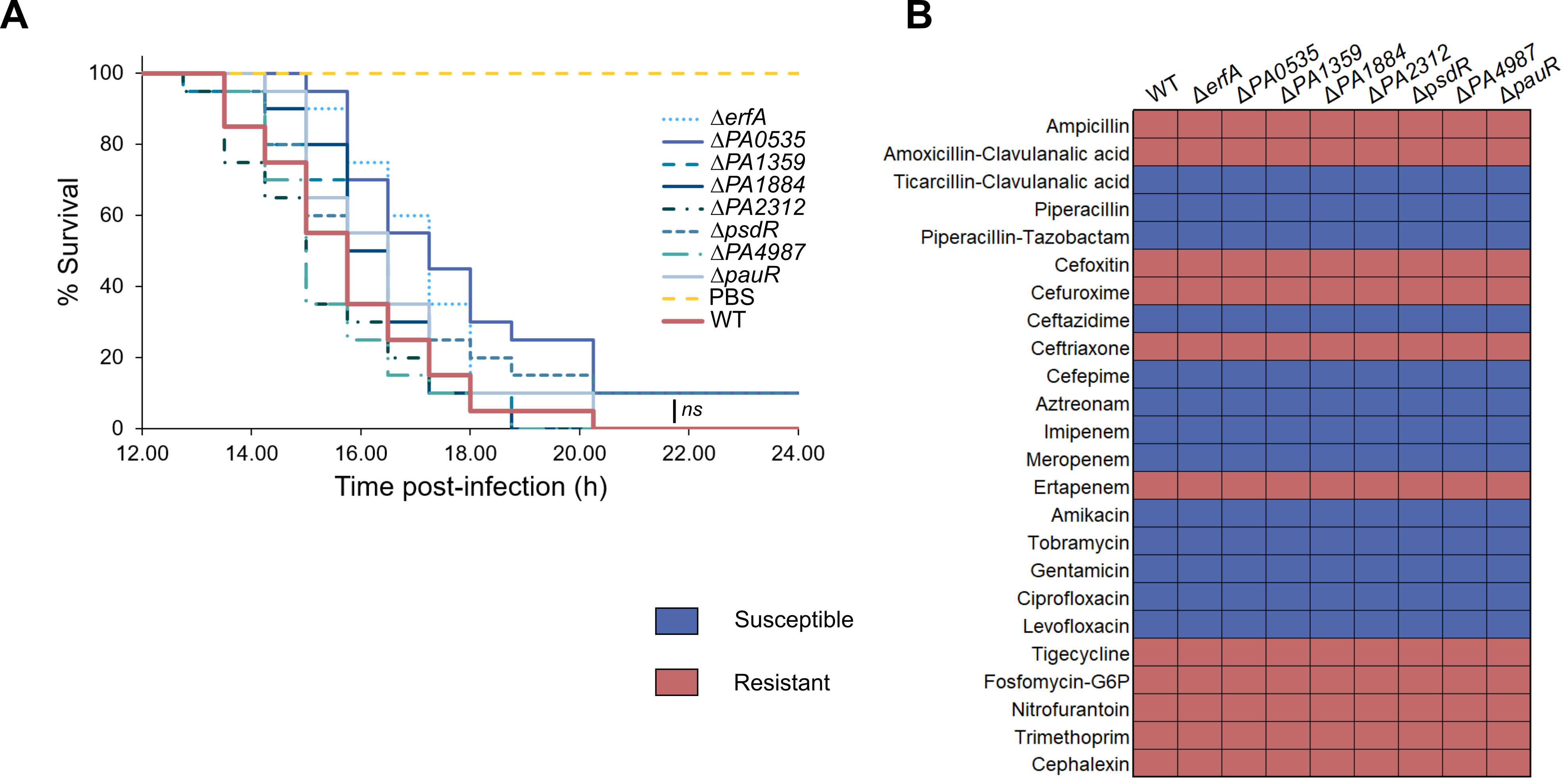

Supplement: FIG S3 [file mSystems.00753-20-sf003.tif]

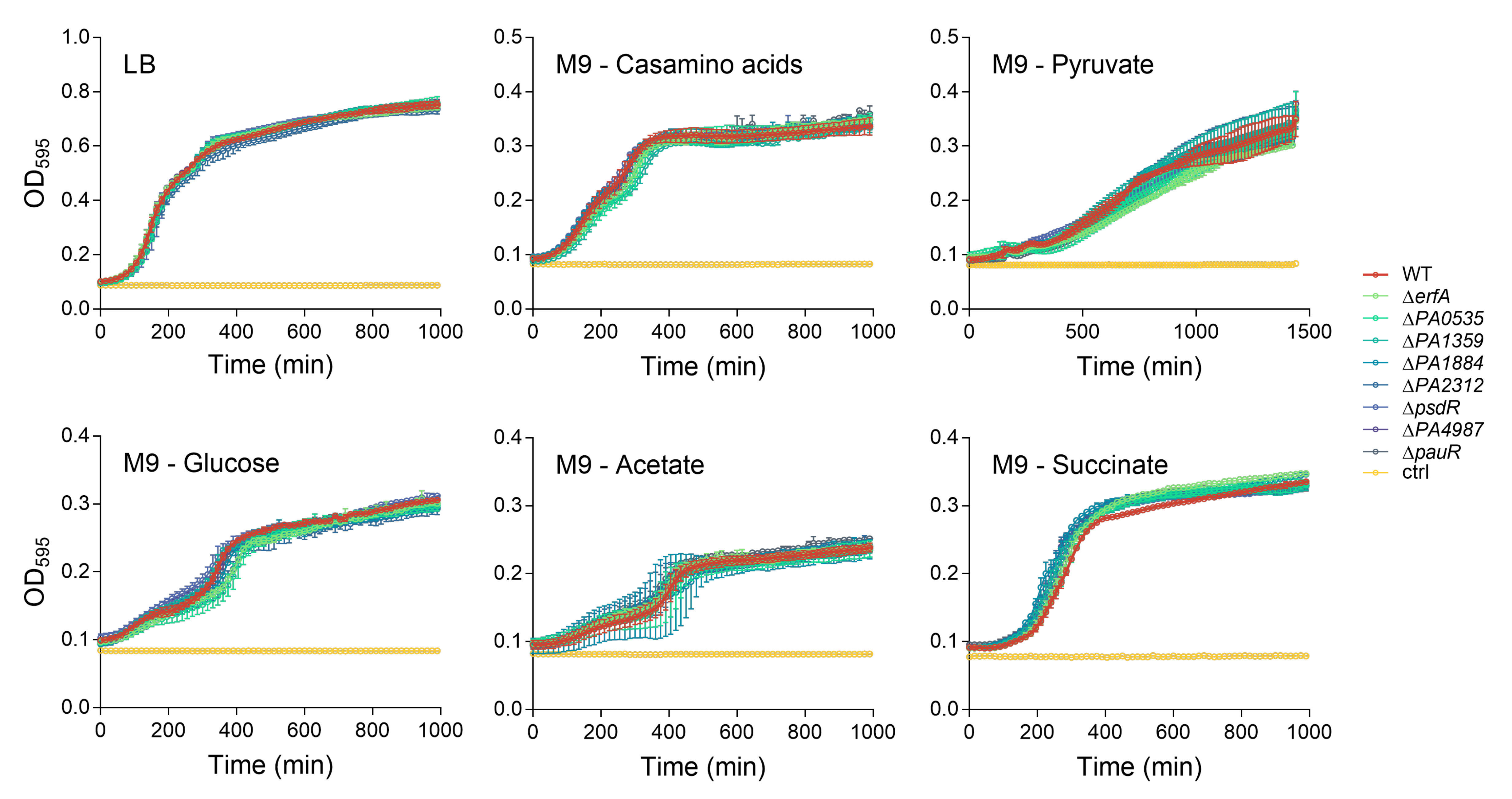

Supplement: FIG S4 [file mSystems.00753-20-sf004.tif]

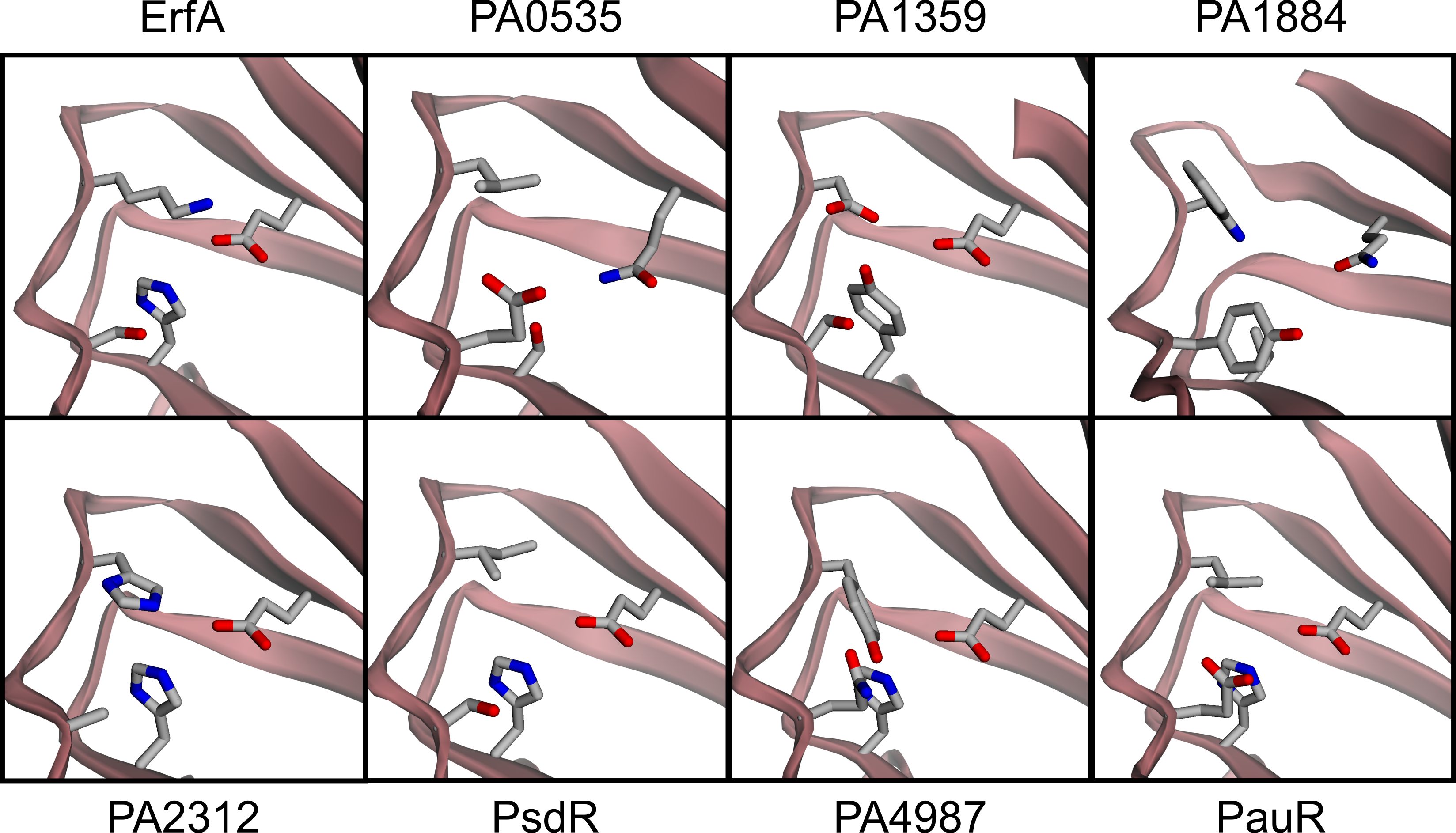

Supplement: FIG S5 [file mSystems.00753-20-sf005.tif]

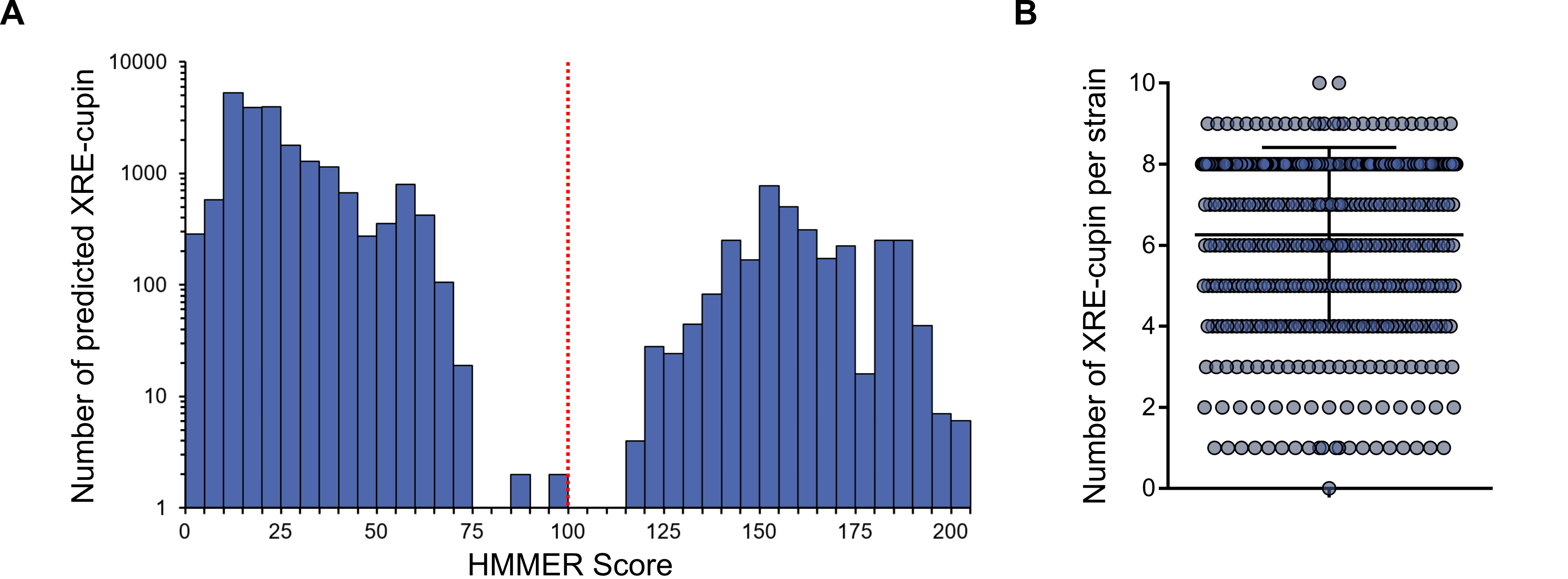

Supplement: FIG S6 [file mSystems.00753-20-sf006.tif]
