# Supplementary material for: Transcription Inhibitors with XRE DNA-Binding and Cupin Signal-Sensing Domains Drive Metabolic Diversification in Pseudomonas
Source: mSystems. 2021 Jan 12;6(1):e00753-20. doi: 10.1128/mSystems.00753-20 (PMC7901475; doi:10.1128/mSystems.00753-20)
Supplement: TABLE S1 [file mSystems.00753-20-st001.pdf]

Strain or plasmid	Genotype or relevant properties	Reference/source
**Strains**		
P. aeruginosa		
PAO1	Wound isolate, sequenced laboratory strain	J. Mougous
PAO1 Δ*erfA*	PAO1 with *erfA* (*PA0225*) deletion	Trouillon *et al.*, *Nucleic Acids Res* 48: 2388-2400, 2020
PAO1 Δ*erfA::erfA*	PAO1 Δ*erfA* with *erfA* in *attB* site	Trouillon *et al.*, *Nucleic Acids Res* 48: 2388-2400, 2020
PAO1 Δ*PA0535*	PAO1 with *PA0535* deletion	This work
PAO1 Δ*PA0535::PA0535*	PAO1 Δ*PA0535* with *PA0535* in *attB* site	This work
PAO1 Δ*PA1359*	PAO1 with *PA1359* deletion	This work
PAO1 Δ*PA1359::PA1359*	PAO1 Δ*PA1359* with *PA1359* in *attB* site	This work
PAO1 Δ*PA1884*	PAO1 with *PA1884* deletion	This work
PAO1 Δ*PA1884::PA1884*	PAO1 Δ*PA1884* with *PA1884* in *attB* site	This work
PAO1 Δ*PA2312*	PAO1 with *PA2312* deletion	This work
PAO1 Δ*PA2312::PA2312*	PAO1 Δ*PA2312* with *PA2312* in *attB* site	This work
PAO1 Δ*psdR*	PAO1 with *psdR* (*PA4499*) deletion	This work
PAO1 Δ*psdR::psdR*	PAO1 Δ*psdR* with *psrD* in *attB* site	This work
PAO1 Δ*PA4987*	PAO1 with *PA4987* deletion	This work
PAO1 Δ*PA4987::PA4987*	PAO1 Δ*PA4987* with *PA4987* in *attB* site	This work
PAO1 Δ*pauR*	PAO1 with *pauR* (*PA5301*) deletion	This work
PAO1 Δ*pauR::pauR*	PAO1 Δ*pauR* with *pauR* in *attB* site	This work
E. coli		
TOP10	Chemically competent cells	Invitrogen
BL21 Star (DE3)	F^- *ompT hsdSB* (rB^- mB^-) *gal dcm rne131*(DE3)	Invitrogen
**Plasmids**		
pRK600	Helper plasmid with conjugative properties (Cm^R)	Kessler *et al.*, *Mol Gen Genet* 233: 293-301, 1992
pEXG2	Allelic exchange vector (Gm^R)	Rietsch *et al.*, *Proc Natl Acad Sci USA* 102: 8006-8011, 2005
pEXG2-mut-PA0535	pEXG2 with SLIC fragment for *PA0535* deletion (Gm^R)	This work
pEXG2-mut-PA1359	pEXG2 with SLIC fragment for *PA1359* deletion (Gm^R)	This work
pEXG2-mut-PA1884	pEXG2 with SLIC fragment for *PA1884* deletion (Gm^R)	This work
pEXG2-mut-PA2312	pEXG2 with SLIC fragment for *PA2312* deletion (Gm^R)	This work
pEXG2-mut-PA4499	pEXG2 with SLIC fragment for *PA4499* deletion (Gm^R)	This work
pEXG2-mut-PA4987	pEXG2 with SLIC fragment for *PA4987* deletion (Gm^R)	This work
pEXG2-mut-PA5301	pEXG2 with SLIC fragment for *PA5301* deletion (Gm^R)	This work
pFLP2	Source of Flp recombinase (Ap^R)	Hoang *et al.*, *Gene* 212: 77-86, 1998
mini-CTX1	Site-specific integrative plasmid (*attP* site, Tc^R)	Hoang *et al.*, *Plasmid* 43: 59-72, 2020
miniCTX-TrrnB-erfA	miniCTX1-TrrnB carrying *erfA* gene (*attP*, Tc^R)	Trouillon *et al.*, *Nucleic Acids Res* 48: 2388-2400, 2020
mini-CTX-PA0535	mini-CTX1 carrying *PA0535* gene (*attP*, Tc^R)	This work
mini-CTX-PA1359	mini-CTX1 carrying *PA1359* gene (*attP*, Tc^R)	This work
mini-CTX-PA1884	mini-CTX carrying *PA1884* gene (*attP*, Tc^R)	This work
mini-CTX-PA2312	mini-CTX1 carrying *PA2312* gene (*attP*, Tc^R)	This work
mini-CTX-PA4499	mini-CTX1 carrying *PA4499* gene (*attP*, Tc^R)	This work
mini-CTX-PA4987	mini-CTX1 carrying *PA4987* gene (*attP*, Tc^R)	This work
mini-CTX-PA5301	mini-CTX1 carrying *PA5301* gene (*attP*, Tc^R)	This work
pET52b	Expression vector (Ap^R)	Novagen
pET52b-PA0535	Expression vector of *PA0535-his10* (Ap^R)	This work
pET52b-PA1359	Expression vector of *PA1359-his10* (Ap^R)	This work
pET52b-PA1884	Expression vector of *PA1884-his10* (Ap^R)	This work
pET52b-PA2312	Expression vector of *PA2312-his10* (Ap^R)	This work
pET52b-PA4499	Expression vector of *PA4499-his10* (Ap^R)	This work
pET52b-PA4987	Expression vector of *PA4987-his10* (Ap^R)	This work
pET52b-PA5301	Expression vector of *PA5301-his10* (Ap^R)	This work
