# Supplementary material for: Transcription Inhibitors with XRE DNA-Binding and Cupin Signal-Sensing Domains Drive Metabolic Diversification in Pseudomonas
Source: mSystems. 2021 Jan 12;6(1):e00753-20. doi: 10.1128/mSystems.00753-20 (PMC7901475; doi:10.1128/mSystems.00753-20)
Supplement: TABLE S2 [file mSystems.00753-20-st002.pdf]

Name	Sequence (5' => 3')	Use
pEXG2-mut-PA0535-sF1	**GGTCGACTCTAGAGGATCCCC**TCGTCCTCGACTACTTCCGC	*PA0535* deletion
pEXG2-mut-PA0535-sR1	**GCGTATACCCAGAGCACCCTG<u>TCA</u>**GTCGTCGGCTACTTCGCTCA	*PA0535* deletion
pEXG2-mut-PA0535-sF2	CAGGGTGCTCTGGGTATACG	*PA0535* deletion
pEXG2-mut-PA0535-sR2	**ACCGAATTCGAGCTCGAGCCC**TCGGTGTCTATGTGGCGGTC	*PA0535* deletion
pEXG2-mut-PA1359-sF1	**GGTCGACTCTAGAGGATCCCC** GGTGCGCACCGAGAGATAGA	*PA1359* deletion
pEXG2-mut-PA1359-sR1	**CTGGCGAAGGCATGGAAGTCG<u>TCA</u>**GCCGGAGACATGTTCGAGGA	*PA1359* deletion
pEXG2-mut-PA1359-sF2	CGACTTCCATGCCTTCGCCA	*PA1359* deletion
pEXG2-mut-PA1359-sR2	**ACCGAATTCGAGCTCGAGCCC**ACGCTGGCTGAAGCCGTAGT	*PA1359* deletion
pEXG2-mut-PA1884-sF1	**GGTCGACTCTAGAGGATCCCC**GTTGGTCGAGGAGCAGTTGC	*PA1884* deletion
pEXG2-mut-PA1884-sR1	**CGTTGCAGGTGGAACGACTGC<u>TCA</u>**GATGCGCGAGATCATCGCCT	*PA1884* deletion
pEXG2-mut-PA1884-sF2	GCAGTCGTTCCACCTGCAAC	*PA1884* deletion
pEXG2-mut-PA1884-sR2	**ACCGAATTCGAGCTCGAGCCC**TGCCTGCTCATGCTGGGGTT	*PA1884* deletion
pEXG2-mut-PA2312-sF1	**GGTCGACTCTAGAGGATCCCC**GCAGGTACGCGAGAGCCTTT	*PA2312* deletion
pEXG2-mut-PA2312-sR1	**TAGAACAGGATCGAGTCGCCG<u>TCA</u>**GACGCGCTGGCCAATCAGAT	*PA2312* deletion
pEXG2-mut-PA2312-sF2	CGGCGACTCGATCCTGTTCT	*PA2312* deletion
pEXG2-mut-PA2312-sR2	**ACCGAATTCGAGCTCGAGCCC**CTGGCGATGATGGGCATGTC	*PA2312* deletion
pEXG2-mut-PA4499-sF1	**GGTCGACTCTAGAGGATCCCC**TCACTGGGGTGCCATTGCAC	*PA4499* deletion
pEXG2-mut-PA4499-sF1	**CAGGCCCATGGTGGTGATGGT<u>TCA</u>**TTCGCTGGCGACTTGGTGCA	*PA4499* deletion
pEXG2-mut-PA4499-sF1	ACCATCACCACCATGGGCCT	*PA4499* deletion
pEXG2-mut-PA4499-sF1	**ACCGAATTCGAGCTCGAGCCC**TAGTCGGTGCCGGTGGTGTA	*PA4499* deletion
pEXG2-mut-PA4987-sF1	**GGTCGACTCTAGAGGATCCCC**GCTGTATGTGCGCGACCAGT	*PA4987* deletion
pEXG2-mut-PA4987-sF1	**GGTGATCACCCAGAGCACCAG<u>TCA</u>**GGTGCCGAGGAAGTGCGTTT	*PA4987* deletion
pEXG2-mut-PA4987-sF1	CTGGTGCTCTGGGTGATCAC	*PA4987* deletion
pEXG2-mut-PA4987-sF1	**ACCGAATTCGAGCTCGAGCCC**TGCTGCTCGGCGATACCATG	*PA4987* deletion
pEXG2-mut-PA5301-sF1	**GGTCGACTCTAGAGGATCCCC** TACAGCACCGAGCGTAGCCA	*PA5301* deletion
pEXG2-mut-PA5301-sF1	**GTGGTGGCGCTGATCAAGCGT<u>TCA</u>**TTGCAGACGAGCACCGACGT	*PA5301* deletion
pEXG2-mut-PA5301-sF1	ACGCTTGATCAGCGCCACCA	*PA5301* deletion
pEXG2-mut-PA5301-sF1	**ACCGAATTCGAGCTCGAGCCC**CTTGCCGACGATGTCTTCGC	*PA5301* deletion
Comp-CTX-PA0535-sF1	**GATATCGAATTCCTGCAGCCC**CAAGGAGATCGCCAAGGAACTG	*PA0535* complementation
Comp-CTX-PA0535-sR1	CAGGAACATCGCGACCTCCGT	*PA0535* complementation
Comp-CTX-PA0535-sF2	**ACGGAGGTCGCGATGTTCCTG**CGTTTGCGCGACCTGTTCTGA	*PA0535* complementation
Comp-CTX-PA0535-sR2	**TCTAGAACTAGTGGATCCCCC**CGACAGGGTAACGCGCCT	*PA0535* complementation

Comp-CTX-PA1359-sF	**GATATCGAATTCCTGCAGCCC**CGAAGAGGATGTACAGCGCCC	*PA1359* complementation
Comp-CTX-PA1359-sR	**TCTAGAACTAGTGGATCCCCC**TCCTCTGGAAGGCTGGCTCGA	*PA1359* complementation
Comp-CTX-PA1884-sF	**GATATCGAATTCCTGCAGCCC**GCTGCCGCTGACCGTGTCCA	*PA1884* complementation
Comp-CTX-PA1884-sR	**TCTAGAACTAGTGGATCCCCC**CCTCGACTGGTCGCCGGAG	*PA1884* complementation
Comp-CTX-PA2312-sF1	**GATATCGAATTCCTGCAGCCC**TGAAGGCGATCTGCTGCGCC	*PA2312* complementation
Comp-CTX-PA2312-sR1	GCGGTCCATGAGGCATTCCTC	*PA2312* complementation
Comp-CTX-PA2312-sF2	**GAGGAATGCCTCATGGACCGC**CGGGCCTGACCGATGAACCT	*PA2312* complementation
Comp-CTX-PA2312-sR2	**TCTAGAACTAGTGGATCCCCC**TCTTCGTCCATGGCAGCAAGG	*PA2312* complementation
Comp-CTX-PA4499-sF	**GATATCGAATTCCTGCAGCCC**TTCCTCCCAGGTGTGGATGGC	*PA4499* complementation
Comp-CTX-PA4499-sR	**TCTAGAACTAGTGGATCCCCC**GATGGGAAGGTGCGCTACGGA	*PA4499* complementation
Comp-CTX-PA4987-sF1	**GATATCGAATTCCTGCAGCCC**CCTCGACCTGGACCAGGTCC	*PA4987* complementation
Comp-CTX-PA4987-sR1	GAAATTCACGGTTGGCGTCCTT	*PA4987* complementation
Comp-CTX-PA4987-sF2	**AGGACGCCAACCGTGAATTTC**AAGGCCGCCTGGAGCATCTGA	*PA4987* complementation
Comp-CTX-PA4987-sR2	**TCTAGAACTAGTGGATCCCCC**CTGGCCCTGCATCGCGACAG	*PA4987* complementation
Comp-CTX-PA5301-sF	**GATATCGAATTCCTGCAGCCC**CGCTACAGCACCGAGCGTAG	*PA5301* complementation
Comp-CTX-PA5301-sR	**TCTAGAACTAGTGGATCCCCC**CTTGCGGCATTAGGTCGCAGC	*PA5301* complementation
pET52b-PA0535-sF	**TTAAGAAGGAGATATACC**ATGAGCGAAGTAGCCGACGA	*PA0535* overexpression
pET52b-PA0535-sR	**CTACCGCGTGGCACCAGAGCGAG**GGCGTATACCCAGAGCACC	*PA0535* overexpression
pET52b-PA1359-sF	**TTAAGAAGGAGATATACC**ATGGAAGAGGTCCGGCAGTG	*PA1359* overexpression
pET52b-PA1359-sR	**CTACCGCGTGGCACCAGAGCGAG**GCTCACCACGTTGCGGGTG	*PA1359* overexpression
pET52b-PA1884-sF	**TTAAGAAGGAGATATACC**ATGGATATCGACGAACTGATTG	*PA1884* overexpression
pET52b-PA1884-sR	**CTACCGCGTGGCACCAGAGCGAG**GTCGTGGAGGATCACCAGC	*PA1884* overexpression

pET52b-PA2312-sF	**TTAAGAAGGAGATATACC**ATGCATACCGAACCCGATGATC	*PA2312* overexpression
pET52b-PA2312-sR	**CTACCGCGTGGCACCAGAGCGAG**GTCCAGGCGCTCCGGGTA	*PA2312* overexpression
pET52b-PA4499-sF	**TTAAGAAGGAGATATACC**ATGACCGTAGACCGCATCGG	*PA4499* overexpression
pET52b-PA4499-sR	**CTACCGCGTGGCACCAGAGCGAG**GGGCGTCGGATGGTCGTC	*PA4499* overexpression
pET52b-PA4987-sF	**TTAAGAAGGAGATATACC**ATGCCCCGCCCGTCACCG	*PA4987* overexpression
pET52b-PA4987-sR	**CTACCGCGTGGCACCAGAGCGAG**GAACGTCGGCGGGGTGATC	*PA4987* overexpression
pET52b-PA5301-sF	**TTAAGAAGGAGATATACC**ATGGACGTCGGTGCTCGTCT	*PA5301* overexpression
pET52b-PA5301-sR	**CTACCGCGTGGCACCAGAGCGAG**GAAATTTGCGGGCGTGGTGG	*PA5301* overexpression
pEXG2-mut-PA0534-BS-sF1	**GGTCGACTCTAGAGGATCCCC**GTTCGACCTCATCTGCCAGG	EMSA (P*PA0534* mut BS)
pEXG2-mut-PA0534-BS-sR1	**GGTTATTTGAGCTCAAAAATAA**CACTTAAAATTTCACGACACCAG	EMSA (P*PA0534* mut BS)
pEXG2-mut-PA0534-BS-sF2	**TTATTTTTGAGCTCAAATAACC**AATATAATTTCACGGAGGTCGCG	EMSA (P*PA0534* mut BS)
pEXG2-mut-PA0534-BS-sR2	**ACCGAATTCGAGCTCGAGCCC**ACGAAGCGTAGCCCCTCGTA	EMSA (P*PA0534* mut BS)
CTX-PA2776-lacZ-sF	**GATATCGAATTCCTGCAGCCC**GGGAAGCGTGAAGAAGTCCG	EMSA (P*PA2776* mut BS)
CTX-PA2776-mut-lacZ-sR	**GCTAGTTAGTTAGGATCCCCC**TGCGGCATCTTGCACCTCGCATTTTT GCTTTTTCAC<br>GATGCCCGGATGCTACCCGA	EMSA (P*PA2776* mut BS)
CTX-mdpA-lacZ-sF	**GATATCGAATTCCTGCAGCCC**CCGCATTGATCAGGTTGCCG	EMSA (P*mdpA* mut BS)
CTX-mdpA-mut-lacZ-sR	**GCTAGTTAGTTAGGATCCCCC**TGTTCTCCTCGTCTGGACCGGGCTGA<br>AATTTAAGTCGAGAACTGATTCAGGTCAAAACAAATTTTCAGT	EMSA (P*mdpA* mut BS)
(Cy5-) pergAB-EMSA-F	(Cy5-) CAGCCTTCTCCCGATGGCAGT	EMSA (Trouillon *et al.*, 2020)
pPA0534-EMSA-F	CAGCCTTCTCCCGATGGCAGT CTCGGGACTGGTGTCGTGAA	EMSA
pPA0534-EMSA-R	CAGCCTTCTCCCGATGGCAGT AGGAACATCGCGACCTCCGT	EMSA
pPA1360-EMSA-F	CAGCCTTCTCCCGATGGCAGT GACATGTTCGAGGACGCTGG	EMSA
pPA1360-EMSA-R	CAGCCTTCTCCCGATGGCAGT CGGGAAACGGGATGTGCACT	EMSA
pPA1885-EMSA-F	CAGCCTTCTCCCGATGGCAGT TATCCATGAGTGGCCCTGGC	EMSA
pPA1885-EMSA-R	CAGCCTTCTCCCGATGGCAGT GGGCAAGCTCCAATGCAAGG	EMSA
pPA2776-EMSA-F	CAGCCTTCTCCCGATGGCAGT CGATTTCGACTCGGGTAGCAT	EMSA
pPA2776-EMSA-2-R	CAGCCTTCTCCCGATGGCAGT TGCGGCATCTTGCACCTCTG	EMSA

pPA4498-EMSA-F	CAGCCTTCTCCCGATGGCAGT CCCGAACCCCTATTTTTAAGTG	EMSA
pPA4498-EMSA-R	CAGCCTTCTCCCGATGGCAGT TGTTCTCCTCGTCTGGACCG	EMSA
pPA4985-6-EMSA-F	CAGCCTTCTCCCGATGGCAGT ACGGGACAGAAACTATCCAGTC	EMSA
pPA4985-6-EMSA-R	CAGCCTTCTCCCGATGGCAGT GCGCTTCAGAAACAGGATATGA	EMSA
uvrD up	CATATCCTGGTGGACGAGTTCC	RT-qPCR
uvrD down	CGCTGAACTGCTGGATGTTCTC	RT-qPCR
qPCR_pauB1_up	ACCTATGGACGGCGATCCTG	RT-qPCR
qPCR_pauB1_down	ACGAAGCGTAGCCCCTCGTA	RT-qPCR
qPCR_fumC1_up2	TCGGGCAACTTCGAACTGAA	RT-qPCR
qPCR_fumC1_down2	GAGCTTGCCCTGGTTGACCT	RT-qPCR
qPCR_PA0265_up2	TGTTCCGCTTCAAGGACGAG	RT-qPCR
qPCR_PA0265_down2	CCATGCCGTACTCCAGTTGC	RT-qPCR
qPCR_PA1360_up	TCCCTGGCGAAAAGCATGT	RT-qPCR
qPCR_PA1360_down	AGGAGCAGCAGCAGGATCAG	RT-qPCR
qPCR_PA1541_up	TCGCCTACGCCCTTTGGGAA	RT-qPCR
qPCR_PA1541_down	TACAGGCCGATGCTTTCGCC	RT-qPCR
qPCR_PA1885_up2	CCGAACGGCTGTACCTGAAG	RT-qPCR
qPCR_PA1885_down2	CAGCCAGGCGCTTGTAGAAA	RT-qPCR
qPCR_PA4498_up2	GCATCAGCACCACCGAAGTC	RT-qPCR
qPCR_PA4498_down2	ACCGAACAGGACGATGCAGA	RT-qPCR
qPCR_PA4500_up	CAACGCCGACGATGTGCTGT	RT-qPCR
qPCR_PA4500_down	TTGTCCAGGCCCATGTCGGT	RT-qPCR
qPCR_PA4985_up	CGTGGTGATGACGTCCACCT	RT-qPCR
qPCR_PA4985_down	ACCACCGCCCAGTAGTCCAT	RT-qPCR
qPCR_PA4986_up	CTGTTCAGCCCCCTGGAAAT	RT-qPCR
qPCR_PA4986_down	GTGGCTCGTTGACCAGGTTG	RT-qPCR
